# CALB2 drives pancreatic cancer metastasis through inflammatory reprogramming of the tumor microenvironment

**DOI:** 10.1186/s13046-024-03201-w

**Published:** 2024-10-03

**Authors:** Jinxin Tao, Yani Gu, Zeyu Zhang, Guihu Weng, Yueze Liu, Jie Ren, Yanan Shi, Jiangdong Qiu, Yuanyang Wang, Dan Su, Ruobing Wang, Yifan Fu, Tao Liu, Liyuan Ye, Wenhao Luo, Hao Chen, Gang Yang, Zhe Cao, Hua Huang, Jianchun Xiao, Bo Ren, Lei You, Taiping Zhang, Yupei Zhao

**Affiliations:** 1 General Surgery Department, State Key Laboratory of Complex Severe and Rare Diseases, Peking Union Medical College Hospital, Chinese Academy of Medical Sciences and Peking Union Medical College, Beijing, 100730 China; 2 Institute of Clinical Medicine, Peking Union Medical College and Chinese Academy of Medical Sciences, Translational Medicine Center, Peking Union Medical College Hospital, Beijing, 100730 China; 3 Biomedical Engineering Facility of National Infrastructures for Translational Medicine, Institute of Clinical Medicine, Peking Union Medical College Hospital, Chinese Academy of Medical Sciences and Peking Union Medical College, Beijing, 100730 China; 4 Department of General Surgery, Pancreatic Disease Center, Ruijin Hospital, Shanghai Jiao Tong University School of Medicine, Shanghai, 200025 China; 5 Department of General Surgery, China‑Japan Friendship Hospital, Beijing, 100029 China

**Keywords:** Pancreatic cancer, Metastasis, Inflammatory reprogramming, Cancer-associated fibroblasts, Organoids, CALB2, CXCL14

## Abstract

**Background:**

Early dissemination to distant organs accounts for the dismal prognosis of patients with pancreatic ductal adenocarcinoma (PDAC). Chronic, dysregulated, persistent and unresolved inflammation provides a preferred tumor microenvironment (TME) for tumorigenesis, development, and metastasis. A better understanding of the key regulators that maintain inflammatory TME and the development of predictive biomarkers to identify patients who are most likely to benefit from specific inflammatory-targeted therapies is crucial for advancing personalized cancer treatment.

**Methods:**

This study identified cell-specific expression of CALB2 in human PDAC through single-cell RNA sequencing analysis and assessed its clinicopathological correlations in tissue microarray using multi-color immunofluorescence. Co-culture systems containing cancer-associated fibroblasts (CAFs) and patient-derived organoids (PDOs) in vitro and in vivo were employed to elucidate the effects of CALB2-activated CAFs on PDAC malignancy. Furthermore, CUT&RUN assays, luciferase reporter assays, RNA sequencing, and gain- or loss-of-function assays were used to unravel the molecular mechanisms of CALB2-mediated inflammatory reprogramming and metastasis. Additionally, immunocompetent KPC organoid allograft models were constructed to evaluate CALB2-induced immunosuppression and PDAC metastasis, as well as the efficacy of inflammation-targeted therapy.

**Results:**

CALB2 was highly expressed both in CAFs and cancer cells and correlated with an unfavorable prognosis and immunosuppressive TME in PDAC patients. CALB2 collaborated with hypoxia to activate an inflammatory fibroblast phenotype, which promoted PDAC cell migration and PDO growth in vitro and in vivo. In turn, CALB2-activated CAFs upregulated CALB2 expression in cancer cells through IL6-STAT3 signaling-mediated direct transcription. In cancer cells, CALB2 further activated Ca^2+^-CXCL14 inflammatory axis to facilitate PDAC metastatic outgrowth and immunosuppression. Genetic or pharmaceutical inhibition of CXCL14 significantly suppressed CALB2-mediated metastatic colonization of PDAC cells in vivo and extended mouse survival.

**Conclusions:**

These findings identify CALB2 as a key regulator of inflammatory reprogramming to promote PDAC metastatic progression. Combination therapy with αCXCL14 monoclonal antibody and gemcitabine emerges as a promising strategy to suppress distant metastasis and improve survival outcomes in PDAC with CALB2 overexpression.

**Supplementary Information:**

The online version contains supplementary material available at 10.1186/s13046-024-03201-w.

## Introduction

Pancreatic ductal adenocarcinoma (PDAC) remains one of the most aggressive and lethal solid malignancies, with a mortality rate nearly equal to its incidence, and it is projected to become the second leading cause of cancer deaths in the United States by 2040 [1, 2]. Only a minority of patients (~ 20%) diagnosed at an early stage is eligible for curative surgical resection in combination with adjuvant chemotherapy [3]. By the time when PDAC is initially diagnosed, the majority of patients have non-resectable locally advanced (~ 30%) or metastatic cancer (~ 50%) [4]. Metastatic spread commonly occurs to the liver, which constitutes the primary cause of PDAC-related mortality [5]. Even though the primary tumor is surgically removed, approximately 70% of patients relapse with hepatic metastasis within 2 years [6]. Therefore, a better understanding of the molecular pathology of metastatic PDAC is crucial for the development of novel therapeutic strategies and the improvement of patient outcomes.

Chronic, dysregulated, persistent and unresolved inflammation is considered as a key characteristic of cancer [7]. Inflammatory programs provide a preferred tumor microenvironment (TME), which functionally cooperate with oncogenic mutations to facilitate tumorigenesis, development and metastasis in most types of cancer [8]. Due to the close relationship between inflammation and tumor progression, targeting inflammation is an important way for improving the efficacy of anti-cancer therapies. However, the pleiotropic nature of inflammatory mediators and their context-dependent roles in cancer underscore the need for precision medicine approaches that consider individual patient characteristics, including the genetic and molecular profiles of tumors, to identify patients who are most likely to benefit from specific cytokine-targeted therapies [9].

PDAC is characterized by a highly heterogeneous, desmoplastic, hypoxic, inflammatory and immunosuppressive TME, which promotes malignant tumor progression and limits therapeutic response to standard regimens based on gemcitabine (GEM) [10–13]. Stromal cells facilitate an inflammatory-fibrotic TME to support the survival and dissemination of cancer cells to the distant organs [14, 15]. Cancer-associated fibroblasts (CAFs), a major cellular constituent of the stroma, play a central role in TME formation and tumor malignancy by shaping the dense extracellular matrix and engaging in cytokine-mediated inflammatory crosstalk with cancer cells [16, 17]. Recent studies on CAF heterogeneity with single cell RNA sequencing (scRNA-seq) technologies have revealed the pleiotropic nature of transcriptionally diverse CAF populations in PDAC, including myofibroblastic CAFs (myCAFs), inflammatory CAFs (iCAFs), and antigen presenting CAF (apCAFs) [18–21]. Stromal-targeted therapies rarely yield promising clinical outcomes and even lead to disease acceleration and the development of more aggressive tumors [22]. This is partially attributed to CAF heterogeneity and highlights the need to further understand their molecular and functional heterogeneity [23].

A fibroblast subtype named FB_CALB2 is identified in high-grade serous tubo-ovarian cancer, which expresses high levels of CALB2, pro-inflammatory cytokines (IL6 and IL18) and fibrosis-promoting genes (COL8A1, CXCL16) [24]. Furthermore, CALB2 (coding for the protein known as calretinin or calbindin 2) is identified as a compartment-specific poor prognostic marker for the tumor area using proteomic analysis of laser-capture microdissected PDAC samples [25]. However, the roles of CALB2 in pancreatic cancer cells and CAFs remain largely elusive. In this study, we first report that CALB2 serves as a key regulator of inflammatory reprogramming to drive PDAC metastasis. Abnormally overexpressed CALB2, either in CAFs or cancer cells, correlates with an unfavorable prognosis and immunosuppressive TME in PDAC patients. In CAFs, CALB2 collaborates with hypoxia to promote the malignant transition from pancreatic stellate cells (PSCs) to iCAFs. In turn, CALB2-activated CAFs upregulate CALB2 expression in cancer cells via IL6-STAT3 inflammatory signaling pathway and support PDAC growth and metastasis. In cancer cells, CALB2-Ca^2+^-CXCL14 axis further promotes an inflammatory and immunosuppressive TME to facilitate PDAC metastasis. Genetic or pharmacologic inhibition of the CXCL14 effectively attenuates the formation of liver metastases in vivo. Collectively, our study uncovers an inflammatory reprogramming mechanism mediated by CALB2 and proposes a novel inflammation-targeting strategy for PDAC patients with CALB2 overexpression.

## Methods

### Human PDAC tissue samples

A total of 36 freshly frozen surgically resected PDAC specimens were collected from patients without preoperative treatment at Peking Union Medical College Hospital (PUMCH). The diagnoses were confirmed by histopathology. Additionally, a tissue microarray (TMA) composed of tumoral and adjacent normal tissues from 190 PDAC patients was obtained to analyze clinicopathological correlation. Detailed clinicopathological features of these patients were listed in Supplementary Table S7. The overall survival data were obtained through electronic medical records or telephone follow-up. This study was approved by the Institutional Ethics Committee of PUMCH (Ethic code: I-22PJ487).

### Multi-color immunofluorescence staining

Multispectral immunofluorescence (IF) staining was performed as previously described [26]. The TMA was incubated with the following antibodies: anti-CALB2 (Abcam, #ab92341, 1:100), anti-FAP (Abcam, #ab314456, 1:100), and anti-CK19 (Abcam, #ab52625, 1:100). Nuclear staining was performed with ProLong Diamond Antifade mounting medium containing DAPI (Invitrogen, #P36971). Whole tissue slide scan was performed on the TissueFAXS Spectra Systems (TissueGnostics GmbH, Vienna Austria). The multispectral image (4-color staining) was separated and the positive cells were divided for analysis of cell number and location distribution, using StrataQuest analysis software (Version 7.1.129, TissueGnostics GmbH, Vienna, Austria).

### Cell lines and culture

Human embryonic kidney 293 T cells (HEK-293 T, CRL-11268, RRID: CVCL_1926), immortalized human normal pancreatic ductal cells (hTERT-HPNE, CRL-4023, RRID: CVCL_C466), and human PDAC cell lines, including AsPC-1 (#CRL-1682, RRID: CVCL_0152), BxPC-3 (#CRL-1687, RRID: CVCL_0186), CFPAC-1 (#CRL-1918, RRID: CVCL_1119), PANC-1 (#CRL-1469, RRID: CVCL_0480), and SW 1990 (#CRL-2172, RRID: CVCL_1723), were obtained from the American Type Culture Collection (ATCC; Rockville, MD, USA). Furthermore, other PDAC cell lines, including HPAC (#IM-H157, RRID: CVCL_3517) and Capan-1 (#IM-H154, RRID: CVCL_0237), were purchased from IMMOCELL (Xiamen, Fujian, China), while HPAF-II (#CL-0611, RRID: CVCL_0313) and SU.86.86 (#CL-0820, RRID: CVCL_3881) were purchased from Pricella Life Science & Technology Co.,Ltd. Immortalized human pancreatic stellate cells (PSCs, #ZQY008) was purchased from ZQXZ-bio (Shanghai, China). All cell lines were validated by short tandem repeat (STR) analysis and tested for mycoplasma contamination.

PDAC cells were cultured in Dulbecco’s modified Eagle’s medium (DMEM), DMEM/Nutrient Mixture F-12 (DMEM/F-12), MEM, RPMI 1640, or IMDM medium supplemented with 5–20% fetal bovine serum (FBS), 1% penicillin/streptomycin, and 1% Normocin™ (Invivogen, #ant-nr-2). PSCs were cultured in human pancreatic stellate cell complete culture medium (ZQXZ-bio, #PCM-H-081). All cells were cultured at 37 °C and 5% CO_2_ and 20% O_2_ in a humidified cell incubator. For hypoxia experiments, cells were cultured in a hypoxic cell incubator at 1% O_2_, 37 °C and 5% CO_2_.

### Construction of stable cell lines

To establish CALB2 knockdown (KD) and CRISPR knockout (KO) metastatic PDAC cells, AsPC-1 and CFPAC-1 were infected with lentivirus carrying shRNAs (vector: hU6-MCS-CBh-IRES-puromycin), and sgRNAs (vector: U6-sgRNA-EF1a-Cas9-FLAG-P2A-puromycin) for 16 h, respectively. Then, 48 h after lentivirus infection, PDAC cells were treated with puromycin for at least 2 weeks. Similarly, to generate CALB2-overexpressing cells, PANC-1, BxPC-3, and PSCs were infected with lentivirus (vector: Ubi-MCS-3FLAG-CBh-IRES-puromycin), while KPC organoids were infected with lentivirus (vector: Ubi-MCS-3FLAG-CBh-gcGFP-IRES-puromycin). For CXCL14 KD, CALB2-overexpressing BxPC-3 cells were infected with lentivirus carrying CXCL14 shRNAs (vector: hU6-MCS-CBh-IRES-Neomycin) and selected with G418. In all cases, the effect of gene knockdown or knockout or overexpression was validated by immunoblotting. All lentiviruses were purchased from GENECHEM (Shanghai, China). The shRNA and sgRNA sequences are listed in Supplementary Table S1-S2.

### CUT&RUN assay

PDAC cells were seeded into 12-well plates and transfected with overexpression plasmids STAT3 using Lipofectamine™ 3000 reagent (Invitrogen, #L3000015) according to the manufacturer’s instructions. The following day, PDAC cells were treated with 100 ng/mL IL6 or PBS. After 48 h, PDAC cells were collected for CUT&RUN experiments using Hyperactive pG-MNase CUT and RUN Assay Kit (Vazyme, #HD101-01, Vazyme Biotech Co.,Ltd). Briefly, 1 × 10^5^ fresh cells were collected and washed once in 100 µL of washing buffer, followed by bound to Concanavalin A (ConA) magnetic beads for 10 min at room temperature (RT). Cells were then treated with anti-STAT3 (Abcam, #ab68153) or IgG antibody at 4 °C overnight. The following day, cells were washed twice with digitonin (DIG) washing buffer and incubated with pG-MNase enzyme at 4 °C for 1 h. After twice DIG washing, cells were incubated with CaCl_2_ at 4 °C for 1 h, followed by incubated with stop buffer at 37 °C for 30 min. Then, beads were gathered, and DNA was eluted from the beads. Finally, qPCR tests were conducted following the manufacturer's recommendations. Primer sequences are listed in Supplementary Table S4.

### Dual-luciferase reporter assay

PDAC cells were seeded into 12-well plates and transfected with overexpression (OE) plasmids STAT3 or the control vector using Lipofectamine™ 3000 reagent (Invitrogen, #L3000015) according to the manufacturer’s instructions. Forty-eight hours later, luciferase reporter plasmids carrying full-length, truncated or mutant CALB2 promoters and renilla luciferase plasmids were co-transfected into PDAC cells. After another 48 h, the firefly and renilla luciferase activities were measured using the Dual Luciferase Reporter Gene Assay Kit (Yeasen, Shanghai, China, #11402ES) and a multifunctional microplate reader (Tristar LB 942, Berthold, Germany) according to the manufacturer’s instructions.

### RNA sequencing and analysis

Total RNA was extracted from PDAC cells or organoids using TRIzol reagent (Thermo Fisher Scientific, #15596018CN). RNA concentration was measured using a Qubit RNA Broad Range kit (Thermo Fisher Scientific, # Q10210). RNA quality was assessed on an Agilent TapeStation 4200 using the Agilent RNA ScreenTape kit (Agilent Technologies, #5067–5576). NEBNext® Ultra™ RNA Library Prep Kit for Illumina (NEB, #E7530L) was used for sequencing library construction, and Illumina NovaSeq platform with PE150 strategy was used for sequencing. The reads were mapped to the GRCh38 human reference genome or the GRCm38 mouse reference genome by HISAT2. Gene expression was quantified using featureCounts. DESeq2 was used to identify differentially expressed genes (DEGs) with adjusted *p* < 0.05 and |log2(fold change)| ≥ 1 or 0.5. Gene set enrichment analysis (GSEA) was performed by ClusterProfler R package (v 4.6.2) depending on the Hallmark gene sets (h.all.v7.4) and the C2 canonical pathway collection (C2.all.v7.4) downloaded from the Molecular Signatures Database (MSigDB) (http://www.gsea-msigdb.org/gsea/msigdb/). Gene set variation analysis (GSVA) was performed using the “gsva’’ method depending on Msigdb Hallmark gene sets (h.all.v7.4) and significantly differential pathways were identified using the limma R package. ClusterProfler R Package v4.0.2 was applied to analyze Gene Ontology (GO) to study the potential functions of each cell subset. The gene ontology of gene sets included molecular function (MF), biological process (BP), and cellular component (CC) categories as references. Significantly enriched pathways were those with adjusted *p* values < 0.05.

### Single-cell RNA-sequencing analysis

The single-cell RNA sequencing (scRNA-seq) analysis of human PDAC samples was based on our previously published dataset (GSA: CRA001160), which included 24 PDAC tumor samples and 11 control pancreases without any treatment. Quality control (QC), dimensional reduction, and clustering were performed with the R package Seurat (v4.3.0.1). The R package GSVA (v1.46.0) was utilized to study the potential functions of each cell subpopulation by calculating their pathway activity depending on Msigdb Hallmark gene sets. Pseudotime trajectory analysis was performed with Monocle 2 (v2.29.0) to map the differentiation of cell subtypes.

### Human and mouse PDAC organoid culture

Primary human PDAC organoids were established form fresh surgically resected human PDAC tissues [27], and mouse PDAC organoids were established from the primary or metastatic pancreatic tumor of KPC mice. Briefly, PDAC tissues were rinsed in cold PBS with penicillin/streptomycin (Gibco, #15140122) for 3 cycles of 5 min each. Subsequently, the tissues were finely minced into fragments and then enzymatic digested on an orbital shaker at 37 °C for 30 min. The digestion medium contained DMEM/F12 medium (Gibco, #11320033), 1 M HEPES (Gibco, #15630130), 1 × GlutaMax (Gibco, #35050061), 100 μg/mL Primocin (InvivoGen, #ant-pm-05), 0.1% bovine serum albumin (BSA, Sigma-Aldrich, #A1933), 10 µM Y-27263 (Selleck, #S1049), 5 mg/mL collagenase II (Worthington, #LS004176), and 100 µg/mL DNase I (Roche, #10104159001). PDAC cell clusters were isolated by centrifugation at 300 g, 4℃ for 5 min and washed twice by cold DMEM/F12 medium. Finally, these cells were seeded into Growth Factor Reduced (GFR) Matrigel (Corning, #356231) in pre-warmed flat bottom cell culture plate and cultured with complete organoid media after incubation in a 37 °C and 5% CO_2_ culture incubator for 15 min. The complete organoid media contained Advanced DMEM/F12 (Gibco, #12634010), medium supplemented with 1 M HEPES, 1 × GlutaMax, 100 μg/mL Primocin, 0.1% BSA, 1 × B27 Supplement (Gibco, #17504044), 10 mM Nicotinamide (Sigma-Aldrich, #72340), 1.25 mM N-acetylcysteine (Sigma-Aldrich, #A9165), 20 ng/mL Wnt-3a (Novoprotein, Shanghai, China, #C06D), 50 ng/mL R-Spondin 1 (Novoprotein, #CX83), 100 ng/mL Noggin (Novoprotein, #CB89), 50 ng/mL epidermal growth factor (EGF, Novoprotein, #C029), 100 ng/mL fibroblast growth factor-10 (FGF-10, novoprotein, #CR11), 10 nM Gastrin I (Sigma-Aldrich, #G9020), 500 nM A83-01 (Selleck, #S7692), 10.5 μm Y-27263 (Selleck, S1049), and 1 μm Prostaglandin E2 (PGE2, Selleck, S3003).

### PDAC organoids and CAFs co-culture model

For the in vitro coincubation organoid growth assay, 1 × 10^5^ PSCs, which were stably transfected with CALB2 OE or control vector, were seeded in the upper chamber of transwell apparatus (0.4 μm insert, Corning), and 5 × 10^4^ cells dissociated from human PDAC organoids were cultured in 50 µL Matrigel with complete organoid media, which were plated in the lower chamber. In the coincubation period of 5 days, PDAC organoid growth was monitored and photographed every 2–3 days.

For the organoid apoptosis assay, 5 × 10^4^ cells dissociated from human PDAC organoids were treated with control (DMSO) or STAT3 inhibitor (stattic, MCE, #HY-13818) and conditioned medium (CM) from PSCs or CAFs for 5 days, and then the organoids were treated with control PBS or GEM for 48 h, followed by assaying organoid apoptosis using the green-fluorescent caspase 3/7 probe reagent (Invitrogen, #R37111). Apoptotic organoids were monitored by fluorescence microscope.

Co-culture assay of PDAC cells and CAFs was described in Supplementary Methods.

### Mouse models of PDAC

We used the following 3 different commonly used mouse models of PDAC. 1) PDAC patient-derived organoids and CAFs orthotopic xenograft (PDOX) mouse model. 1 × 10^6^ patient-derived PDAC organoids mixed with 5 × 10^5^ CAFs in 50 µL of 50% Matrigel (Corning, #356231) were injected orthotopically into the pancreas of female 6–8 week-old immunodeficient NSG mice (Jackson Laboratories). 2) Human PDAC cell lines splenic xenograft mouse model. 1 × 10^6^ human PDAC cell lines in 50 µL of PBS were injected into the spleen of female 6–8 week-old immunodeficient NSG mice. 3) The KPC (LSL-K-Ras^LSLG12D/+^; LSL-p53^R172H/+^; Pdx1-Cre) genetically engineered mouse-derived organoids splenic allograft mouse model. Mouse PDAC organoids were established from KPC mouse pancreatic tumor tissues, followed by injecting 5 × 10^5^ mouse PDAC organoids in 50 µL PBS into the spleen of female 6–8 week-old syngeneic immunocompetent C57BL/6 mice (Jackson Laboratories).

The animal experiments were performed based on protocols approved by the Animal Ethics Committee of Peking Union Medical College Hospital (approval number: XHDW-2022-00). More details of the animal experiments were described in Supplementary Methods.

### Statistical analysis

The statistical analyses were performed using SPSS version 22.0 (IBM Corp. Armonk, NY, USA), Prism Software (GraphPad Software Inc.) and R 4.2.1 (https://www.R-project.org/). All results were presented as mean ± the standard deviation (SD) from no less than three independent experiments, unless otherwise noted. Comparison of group difference(s) was performed using student’s t-test or one-way ANOVA. Non-parametric tests, including Mann-Whitney U or Kruskal-Wallis H test, were used for non-normal distributions or variance homogeneity. The Kaplan-Meier method was applied for survival analysis and the log-rank test was used to compare the survival curves. *P* < 0.05 was considered statistically significant.

## Results

### CALB2 is overexpressed both in CAFs and cancer cells and correlates with immunosuppressive TME in human PDAC

**Fig. 1:**
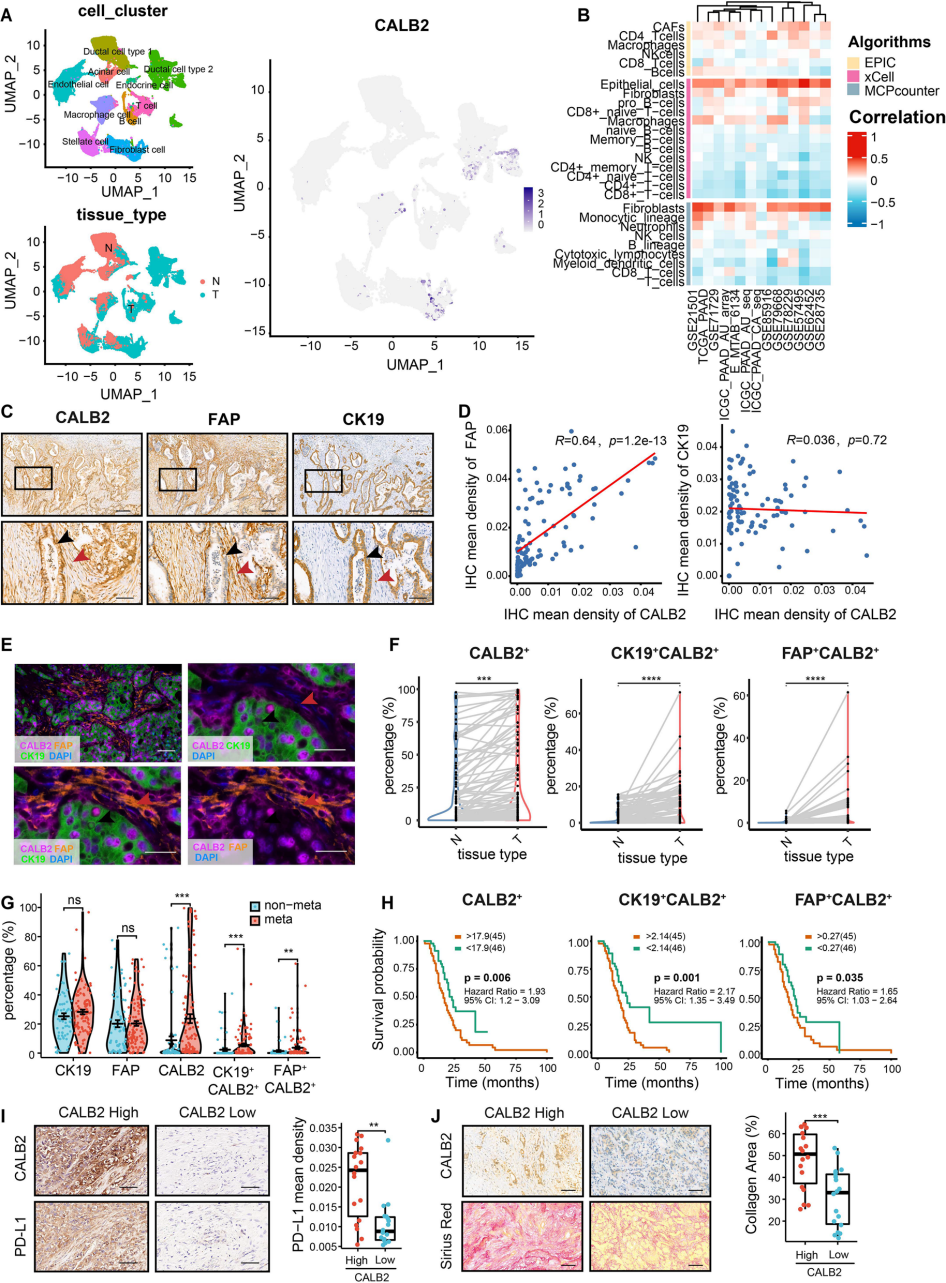
CALB2 is overexpressed both in CAFs and cancer cells and correlates with immunosuppressive TME. (**A**) UMAP plots of single-cell transcriptome data identified cell-specific expression of CALB2 in human PDAC tissues. (**B**) Heatmap illustrating the correlations of CALB2 expression with cell infiltration in the TME across multiple PDAC datasets. (**C**) Representative IHC staining of CALB2, FAP, and CK19 in human PDAC tissues. Black arrows indicate CALB2^+^CK19^+^FAP^−^ cancer cell and red arrows indicate CALB2^+^CK19^–^FAP^+^ CAF. Scale bars, 200 μm (top), 50 μm (bottom). (**D**) Scatter plot demonstrating the correlation between the IHC mean density of CALB2 and FAP or CK19. (**E**) Tissue-based cyclic immunofluorescence for CALB2 (pink), FAP (orange), CK19 (green), in human PDAC tissues. Scale bars, 50 μm (top left), 20 μm (top right and bottom). (**F**) Comparison of the indicated cell types in matched cancer and adjacent tissues. (**G**) Comparison of the indicated cell types in tumors without or with metastasis. (**H)** Human PDAC tissues were classified into CALB2-High or CALB2-Low groups (left), cancer cell CALB2-High or CALB2-Low groups (middle), or CAFs CALB2-High or CALB2-Low groups (right), based on the cyclic immunofluorescence, followed by examining patient overall survival using Kaplan-Meier survival analysis. (**I)** Representative CALB2 and PD-L1 IHC staining in human PDAC tissues, and quantification of PD-L1 positive cells area per field (*n* = 36). Scale bars, 100 μm. (**J**) CALB2 IHC and Sirius Red staining in human PDAC tissues and quantification of collagen deposition using Sirius Red staining (*n* = 36). Scale bars, 50 μm. Error bars, mean ± SD; *p < 0.05, **p < 0.01, ***p < 0.001, ****p < 0.0001; ns, not significant; by Pearson’s correlation test (**D**), paired t test (**F**), Student’s t test (**G**, **I**, and **J**) or log-rank test (**H**)

To unravel the intratumoral heterogeneity of CAFs in human PDAC, we re-analyzed previously generated single-cell RNA sequencing (scRNA-seq) profiles from PDAC tumor samples and control pancreases. Inspired by Siel Olbrecht et al.’s report on a fibroblast subtype named FB_CALB2 [24], we noticed a CAF subset characterized by high expression of CALB2 (Fig. 1A). In addition to CAFs, CALB2 was also highly expressed in malignant ductal cells within the tumor tissue (Fig. 1A, S1A). Based on correlation analysis using TCGA pan-cancer transcriptome data, we observed a positive correlation between CALB2 expression and CAF infiltration across nearly all tumors (Fig. S1B). Conversely, CALB2 expression showed a negative correlation with CD8^+^ T cell infiltration (Fig. S1C). These findings were confirmed in multiple PDAC datasets (Fig. 1B). Consistent with the transcript level, the abundance of CALB2 protein in human PDAC specimens showed a positive correlation with most classical markers of CAFs and epithelial cells, but a negative correlation with CD8 (Fig. S2A-C).

Next, we evaluated CALB2 expression in tumor tissues surgically resected from 36 PDAC patients and its relationship with TME changes using immunohistochemistry (IHC). CALB2 was overexpressed in tumor stromal CAFs, where the mean density of IHC showed a strong positive correlation between CALB2 and fibroblast activation protein (FAP) (Fig. 1C-D). Furthermore, CALB2 was also overexpressed in cancer cells (Fig. 1B-C), but no significant correlation was observed in the mean density between CALB2 and CK19 (Fig. 1D). To enhance data credibility, we used a tissue microarray (TMA) cohort containing 190 PDAC patients and conducted multi-color immunofluorescence staining to confirm the correlation between CALB2 and stromal CAFs and cancer cells (Fig. 1E, Supplementary Table S7). In 138 pairs of matched cancer and adjacent tissues, the proportion of CALB2^+^, CK19^+^CALB2^+^, and FAP^+^CALB2^+^ cells significantly increased in tumor tissues (Fig. 1F). Consistently, both the RNA and protein abundance of CALB2 was significantly higher in tumor tissues compared to normal adjacent tissues in multiple PDAC datasets (Fig. S1D-E). By classifying our cohort into metastasis (spread to lymph node or distant organs) and non-metastasis groups based on their American Joint Committee on Cancer (AJCC) N and M staging, we found that CALB2^+^ cancer cells and CALB2^+^ CAFs were notably more prevalent in tumor with metastasis, indicating the potential of CALB2 to promote PDAC metastasis (Fig. 1G). Additionally, CALB2 overexpressed in cancer cells and CAFs both strongly correlated with a reduced median overall survival (OS) of PDAC patients (Fig. 1H). Comprehensive Cox regression analysis for various survival outcomes, including OS, disease-free survival (DFS), relapse-free survival (RFS), disease-specific survival (DSS), and progression-free survival (PFS), across multiple PDAC transcriptome datasets (Fig. S3A), showed that CALB2 was an unfavorable prognostic factor, which was coincident with survival analysis result at the CALB2 protein level (Fig. S3B).

To ask whether CALB2 overexpression is associated with the immunosuppressive TME, we first analyzed the correlations between CALB2 and the steps of the cancer immunity cycle [28]. CALB2 negatively correlated with the critical steps of the cancer-immunity cycle, including priming and activation (Step 3), trafficking of immune cells to tumors (Step 4), infiltration of immune cells into tumors (Step 5), recognition of cancer cells by T cells (Step 6), and killing of cancer cells (Step 7) (Fig. S3C). Then, we calculated the correlations between CALB2 and the predicted ICB response signatures. CALB2 negatively correlated with the enrichment scores for the majority of immunotherapy-related positive signatures (Fig. S3D) [29]. Furthermore, CALB2 was positively correlated with immune checkpoints in multiple PDAC datasets, such as PD-L1 and PD-L2 (Fig. S3E). Next, we evaluated collagen deposition and tumor-infiltrating immune cell populations in our cohort. CALB2 overexpression in human PDAC tissues significantly correlated with an increase in PD-L1 expression and collagen deposition (Fig. 1I-J). Altogether, CALB2 is overexpressed in both cancer cells and CAFs in human PDAC, and correlates well with the immunosuppressive and desmoplastic TME and poor survival.

### CALB2 collaborates with hypoxia to activate an inflammatory fibroblast phenotype and enhances PDAC migration and growth

**Fig. 2:**
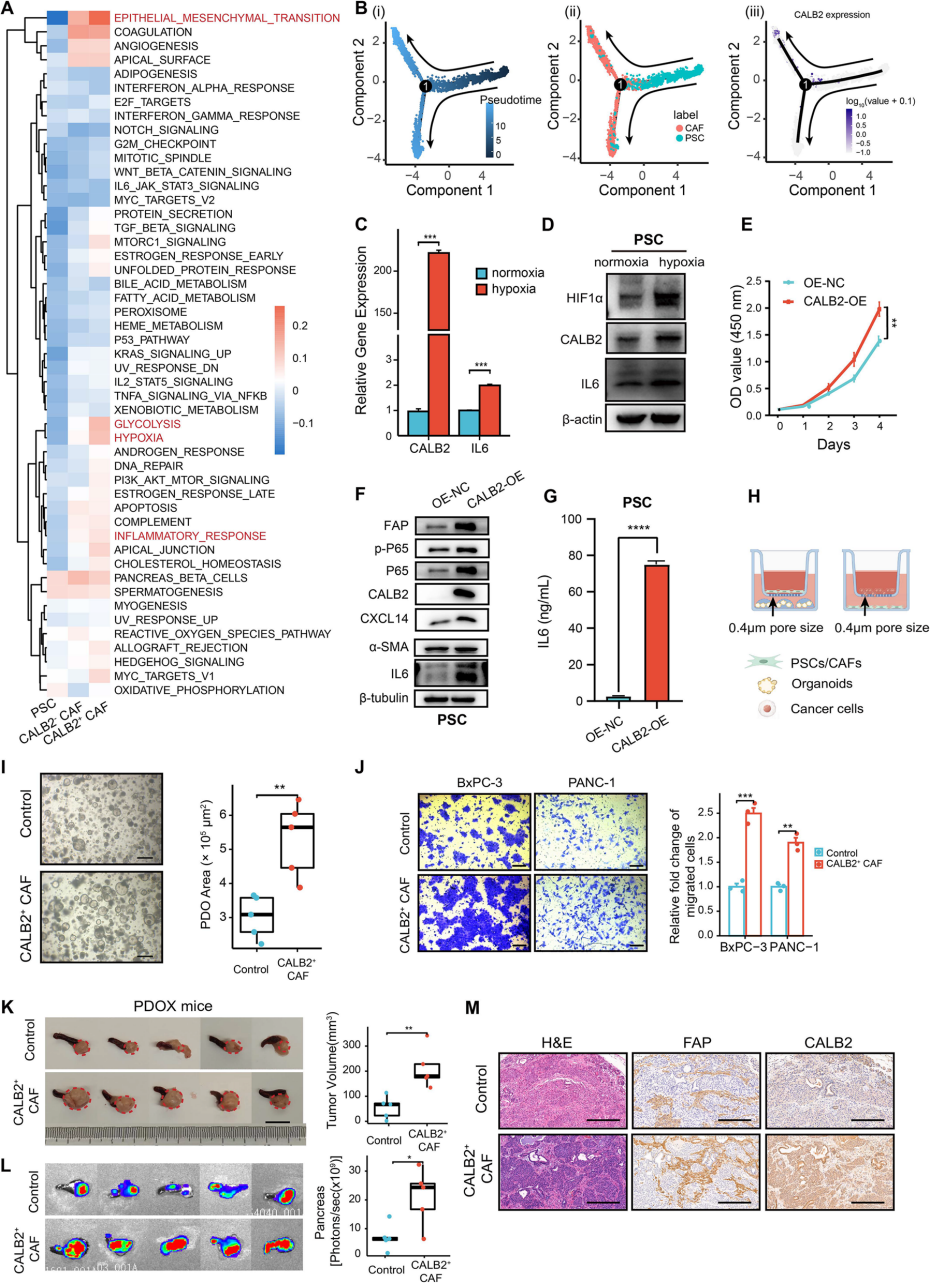
CALB2 collaborates with hypoxia to activate an inflammatory fibroblast phenotype and enhances PDAC migration and growth. (**A**) Heatmap illustrating the gene set variation analyses (GSVA) score of the indicated CAF subpopulations using human scRNA-seq data. (**B**) Deciphering the differentiating signatures by trajectory inferences. (i-ii) Cell trajectory of PSCs, CALB2^−^ and CALB2^+^ CAF subpopulations. (iii) CALB2 expression in the process of CAF differentiation. Black arrows indicate the direction of pseudotime in trajectory plot. (**C-D**) PSCs were exposed to normoxia (20% O_2_) or hypoxia (1% O_2_) for 72 h, followed by RT-qPCR analysis (**C**) and western blotting (**D**). (**E**-**G**) PSCs were subjected to stable CALB2 overexpression (CALB2-OE), followed by examining cell growth using proliferation assay (**E**), the indicated proteins using western blotting (**F**), and IL6 production using ELISA (**G**). (**H**) Schematic diagram showing the establishment of the transwell co-culture system of organoid growth (left) or cell migration (right). (**I**) Transwell co-culture assays of growth of PDAC patient-derived organoids (PDOs) co-cultured with Control or CALB2^+^ CAFs for 5 days. (**J**) Transwell co-culture assays of migration of BxPC-3 and PANC-1 cells co-cultured with Control or CALB2^+^ CAFs for 24 h. (**K-M**) PDAC PDOs were orthotopically transplanted with Control or CALB2^+^ CAFs into the pancreas of NSG mice for 5 weeks (*n* = 5), followed by examining tumor growth (**K**), ex vivo pancreas imaging (**L**), representative H&E staining, and IHC of FAP and CALB2 (**M**). Scale bars, 500 μm (**I**), 100 μm (**J**), 1 cm (**K**), and 200 μm (**M**). Error bars, mean ± SD; *p < 0.05, **p < 0.01, ***p < 0.001, ****p < 0.0001; ns, not significant; by Student’s t test

To figure out the cellular functions of CALB2 overexpression in CAFs in human PDAC, we preformed pathway enrichment analysis using hallmark signatures from the MSigDB to identify the heterogeneity between CALB2^+^ CAF (CALB2-overexpressing or CALB2-activated CAF) and CALB2^−^ CAF (CAF without CALB2 expression) subpopulations in our human PDAC scRNA-seq cohort. PDAC CAF precursor cells-pancreatic stellate cells (PSCs)-were also included in the analysis [30]. Epithelial-mesenchymal transition (EMT), glycolysis, hypoxia, and inflammatory response pathways were notably enriched in CALB2^+^ CAFs compared to CALB2^−^ CAFs and PSCs (Fig. 2A). These pathways were also enriched in PDAC tissues with high expression of CALB2 (Fig. S4A-B). Next, pseudo-time analysis was performed on PSC and CAF subpopulations. The cell trajectory of PSCs showed a tendency to differentiate into two CAF subpopulations, with one of them upregulating CALB2 expression during the process of PSC differentiation (Fig. 2B). Thus, we hypothesized that CALB2^+^ CAFs were derived from PSCs under specific conditions in the TME. The TME of PDAC is characterized by high levels of hypoxia due to dysregulated vascularization and increased demand for cancer cell growth [12]. Hypoxic culture conditions result in an iCAF phenotype in PSCs, as well as enrichment of an inflammatory response signature and IL6/JAK/STAT signaling [31]. To test our hypothesis, we exposed immortalized PSCs to normoxic (20% O_2_) or hypoxic (1% O_2_) conditions for 72 h. Both CALB2 and IL6 expressions were significantly increased in hypoxia (Fig. 2C-D). Then, we constructed PSCs stably overexpressing CALB2 (CALB2-OE) and found that they proliferated better than the control (Fig. 2E). The expression of CAF markers, especially FAP and IL6, was significantly elevated (Fig. 2F-G). These results indicated that CALB2 may collaborates with hypoxia to activate an iCAF phenotype.

FAP^+^ CAFs and iCAFs are generally considered pro-tumorigenic and represent a valuable therapeutic target [32]. A continuous crosstalk between CAFs and tumor cells facilitates the growth and metastasis of PDAC [33]. Ligand-receptor-based cancer-immune crosstalk analysis demonstrated that stellate cells, fibroblast cells, and ductal cells ranked high among all upregulated cross-talking cell types in CALB2-high expression cells compared to CALB2-low expression cells, suggesting that CALB2 may engage in cell-cell communication between PSCs, CAFs, and cancer cells (Fig. S4C-D). To address whether CALB2 overexpression in CAFs affects cancer cells and given the capability of CALB2^+^ CAFs to secrete inflammatory cytokines, we first indirectly co-cultured human PDAC organoids with CALB2-OE PSCs (as a substitute for CALB2^+^ CAFs) or OE-NC PSCs (Control) to examine the effects of CAF-derived soluble factors on PDAC organoids (Fig. 2H). Compared to the control, CALB2^+^ CAFs significantly promoted PDAC organoid growth from single cells (Fig. 2I). Due to the difficulty to detect and quantify the migration of organoids, we then indirectly co-cultured human primary pancreatic cancer cell lines with CALB2-OE PSCs to investigate the impact of CALB2^+^ CAFs on cancer cell migration (Fig. 2H). The results demonstrated that CALB2^+^ CAFs effectively enhanced the migratory capacity of PDAC cells (Fig. 2J). Finally, to explore whether CALB2 is essential for CAFs to fuel tumor growth in vivo, we established patient-derived organoid xenograft (PDOX) mouse models using human PDAC organoids that overexpressed luciferase, co-injected with control or CALB2^+^ CAFs. Compared to the control, CALB2^+^ CAFs significantly promoted cancer cell proliferation and tumor growth in vivo (Fig. 2K-L). Taken together, CALB2 plays a crucial role in the activation of CAFs and their crosstalk with cancer cells, ultimately leading to the increased growth and malignancy of PDAC.

### CALB2 is upregulated in PDAC cells through IL6-STAT3 inflammatory signaling pathway

**Fig. 3:**
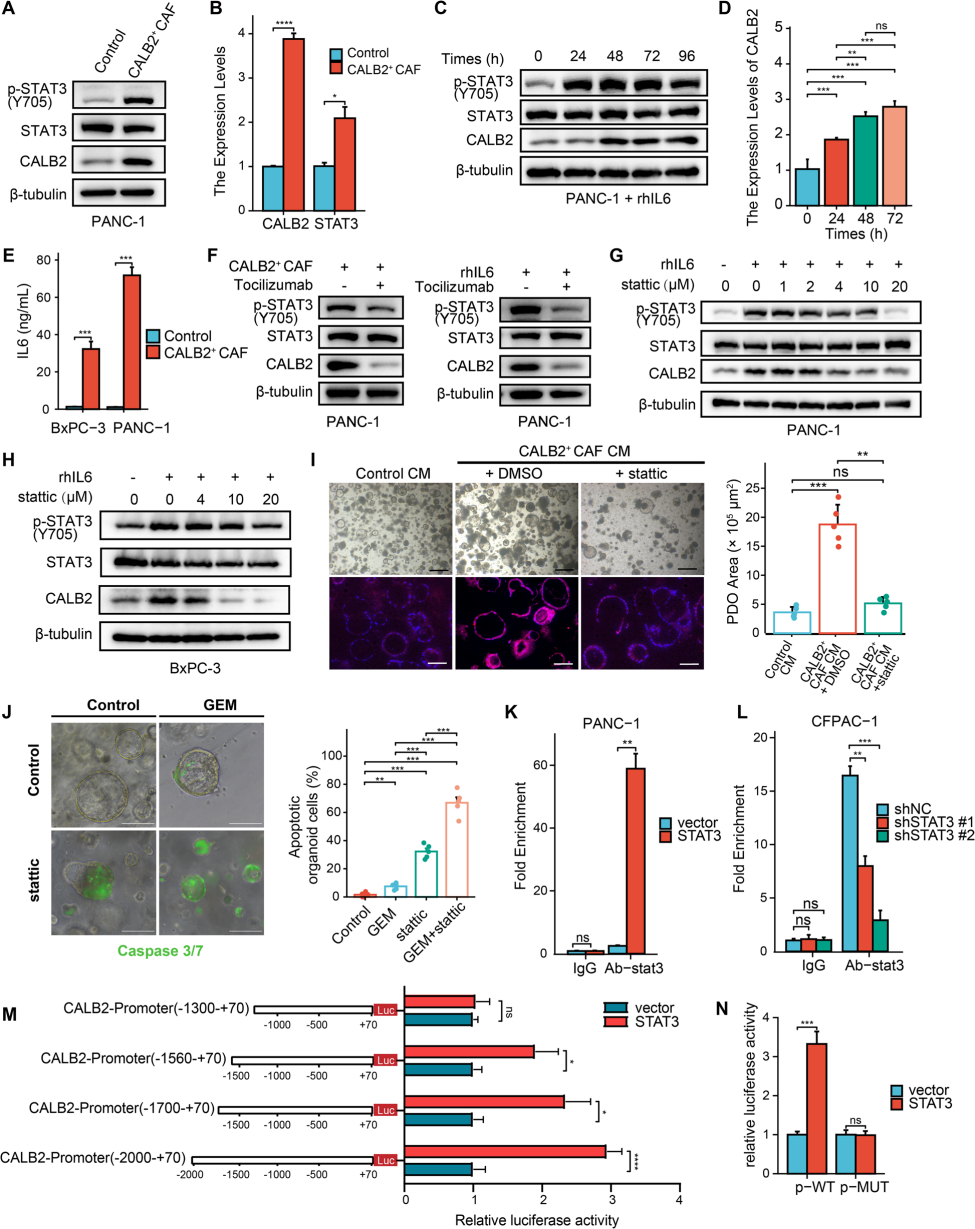
CALB2 is upregulated in PDAC cells through IL6-STAT3 inflammatory signaling pathway. (**A-B**) PANC-1 cells were co-cultured with CALB2^+^ CAFs or control CAFs for 72 h, followed by western blotting (**A**) and RT-qPCR analysis (**B**). (**C-D**) PANC-1 cells were treated with 100 ng/mL rhIL6 over the time, followed by western blotting (**C**) and RT-qPCR analysis (**D**). (**E**) Measurement of IL6 concentration in the co-culture system containing CALB2^+^ CAFs or control CAFs using ELISA. (**F**) PANC-1 cells were pre-treated with 2.5 μg/mL Tocilizumab (IL6R neutralizing antibody) for 24 h, then cultured with CALB2^+^ CAFs or treated with 100 ng/mL rhIL6 for 48 h, followed by western blotting. (**G-H**) PANC-1 (**G**) or BxPC-3 (**H**) cells were treated with the indicated concentration of stattic (STAT3 inhibitor) and 100 ng/mL rhIL6 for 48 h, followed by western blotting. (**I**) Human PDAC organoids were dissociated into single cells and cultured with the following conditions for 5 days: control CAFs conditioned medium (CM) + DMSO (Control), CALB2^+^ CAFs CM + DMSO, and CALB2^+^ CAFs CM + 4 μΜ stattic, followed by microscopic imaging (top left), organoid tissue-based cyclic immunofluorescence to examine CALB2 expression (bottom left) and quantification of organoid growth (right). (**J**) Human PDAC organoids were dissociated into single cells and cultured with CALB2^+^ CAFs CM + 4 μM stattic or control DMSO for 5 days, and then were treated with control PBS or GEM for 48 h, followed by assaying organoid apoptosis and quantification. (**K**-**L**) CUT&RUN-qPCR assays showing binding of STAT3 to CALB2 promoter region was significantly enhanced by STAT3 overexpression in PANC-1 cells (**K**), while it was substantially impaired by STAT3 knockdown in CFPAC-1 cells (**L**). (**M**) Sequential deletions for evaluating the transcriptional activity of the CALB2 promoter in PANC-1 cells with or without STAT3 overexpression. (**N**) Relative luciferase activity of luciferase reporter plasmids bearing wild-type (WT) or mutant (MUT) CALB2 promoter in control or STAT3-overexpressing PANC-1 cells. Scale bars, 500 μm (**I** top), 100 μm (**I** bottom and **J**). Error bars, mean ± SD; *p < 0.05, **p < 0.01, ***p < 0.001, ****p < 0.0001; ns, not significant; by one-way ANOVA (**D**, **I**, **J**, and **L**) or Student’s t test (**B**, **E**, **K**, **M**, and **N**)

In the PDOX model co-transplanted with human PDAC organoid and CALB2^+^ CAFs, we observed that the IHC intensity of CALB2 is not only much higher in stromal CAFs than in the control, but also in cancer cells (Fig. 2M). Hence, we reasoned that CALB2 overexpression in cancer cells might correlate with the ability of CALB2^+^ CAFs to act on cancer cells. Given the notably increased secretion of IL6 in CALB2^+^ CAFs (Fig. 2G), we examined the effect of CALB2^+^ CAFs on the STAT3 signaling pathway in human primary PDAC cells in vitro. After indirect co-culture with CALB2^+^ CAFs for 72 h, the STAT3 pathway was activated and CALB2 expression was elevated in PDAC cells (Fig. 3A-B, Fig. S5A-B). To investigate whether IL6 activates CALB2 expression, PDAC cells were treated with recombinant human IL6 (rhIL6, proteintech, #HZ-1019). Western blotting assays showed that the levels of phospho-STAT3 and CALB2 were both markedly elevated in PDAC cells over the treatment time, similar to those co-cultured with CALB2^+^ CAFs (Fig. 3C-D, Fig. S5C-D). Moreover, the secretion of IL6 was significantly increased in the co-culture system with CALB2^+^ CAFs compared to control CAFs (Fig. 3E). This effect was abolished when Tocilizumab (Selleck, #A2012), a monoclonal anti-human interleukin-6 receptor (IL6R) neutralizing antibody that prevents the binding of IL6 to the IL6R, was added to the co-culture or rhIL6 treatment system (Fig. 3F, Fig. S5E-F), suggesting that IL6 played a pivotal role in CALB2^+^ CAFs-induced CALB2 expression in PDAC cells. Next, to investigate whether IL6-dependent STAT3 activation is essential for CALB2 expression in cancer cells, various concentrations of stattic (MCE, #HY-13818), a potent STAT3 inhibitor, were added to the cancer cells with rhIL6 treatment. Low concentrations of stattic attenuated IL6-induced phospho-STAT3 activation and CALB2 upregulation in PDAC cells, while high concentrations of stattic totally abolished the effect, similar to the levels observed without rhIL6 addition (Fig. 3G-H). After stattic treatment, both the growth and CALB2 expression were decreased in human PDAC organoids cultured with conditioned medium (CM) from CALB2^+^ CAFs (Fig. 3I). Additionally, the sensitivity of PDAC organoids to gemcitabine mediated apoptosis was increased with stattic treatment (Fig. 3J). These findings suggested that CALB2^+^ CAFs promote CALB2 expression in cancer cells, presumably by secreting IL6 to activate STAT3 signaling pathway.

The results above showed that co-culture with CALB2^+^ CAFs or treatment with rhIL6 resulted in a significant increase in both CALB2 protein and mRNA expression in cancer cells (Fig. 3A-D, Fig. S5A-D). Given the role of STAT3 as a transcription factor has been well documented, we ask whether STAT3 directly participates in the transcription of CALB2. PDAC cell lines were transfected with STAT3 or shSTAT3 plasmids, treated with rhIL6, and then harvested for CUT&RUN-qPCR. The enrichment of STAT3 on the CALB2 promoter in PDAC cells significantly increased after STAT3 overexpression (Fig. 3K), while the enrichment significantly decreased after STAT3 knockdown (Fig. 3L). Luciferase reporter assays further showed that the reporter expression levels were significantly increased in STAT3-overexpressing PDAC cells (Fig. 3M). Based on the predicted binding sites in the CALB2 promoter region (Supplementary Table S6), we constructed a series of luciferase reporter plasmids containing different lengths of the CALB2 promoter sequences and found that the -1560 bp to -1300 bp region of the CALB2 promoter was the core element required for STAT3-induced transcriptional activation of CALB2 (Fig. 3M). Furthermore, the mutation of the binding sequence ATGCCTGTAAT almost completely abolished the STAT3 overexpression-mediated increase in luciferase activity (Fig. 3N), further confirming the gene transcription effect of the specific binding of STAT3 to the CALB2 promoter. These findings suggested that IL6-activated STAT3 directly binds to the CALB2 promoter and regulates its transcription. Overall, our results demonstrated that IL6-STAT3 signaling-mediated direct transcription plays a crucial role in upregulating CALB2 expression in PDAC cells.

### Downregulation of CALB2 inhibits epithelial-mesenchymal transition and metastatic outgrowth of PDAC

**Fig. 4:**
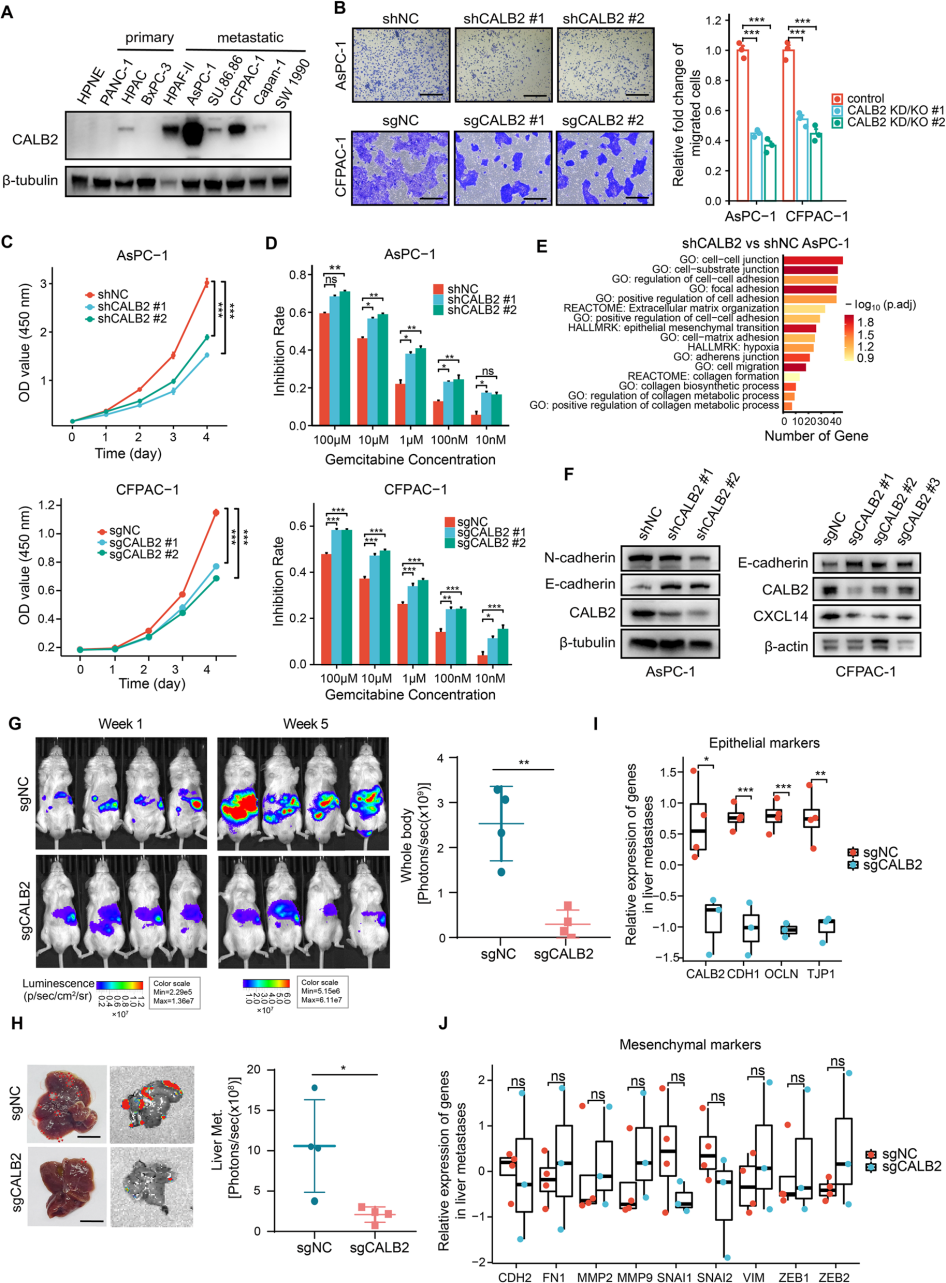
Downregulation of CALB2 inhibits epithelial-mesenchymal transition and metastatic outgrowth of PDAC. (**A**) Western blotting of CALB2 expression in human normal pancreatic ductal cells (HPNE), and human primary and metastatic PDAC cell lines. (**B**) The migration ability of control, or CALB2 KD or KO PDAC cells was determined by Transwell assays. (**C-D**) The influence of CALB2 knockdown (KD) or knockout (KO) on the in vitro proliferation (**C**) and sensitivity to gemcitabine (**D**) of PDAC cells was evaluated by CCK8 assays. (**E**) Significantly downregulated pathways (FDR < 0.05 and logFC > 0.5) identified by DAVID analysis of CALB2-KD AsPC-1 cells (*n* = 3) compared to control AsPC-1 cells (*n* = 3). (**F**) Western blot analysis of E-cadherin and N-cadherin in CALB2-KD AsPC-1 stable cell lines (left) or CALB2-KO CFPAC-1 stable cell lines (right). (**G-H**) IVIS living imaging (**G**), representative bright-field images of liver tissues and ex vivo liver imaging (**H**) after 5 weeks of splenic injection of CALB2-KO or control CFPAC-1 stable cell lines (*n* = 4 per group). (**I-J**) Relative expression of epithelial (**I**) and mesenchymal (**J**) genes in liver metastases from CALB2-KO (*n* = 3) and control (*n* = 4) tumors using RT-qPCR (2^−Δt^). Scale bars, 200 μm (**B**), 1 cm (**H**). Error bars, mean ± SD; *p < 0.05, **p < 0.01, ***p < 0.001, ****p < 0.0001; ns, not significant; by one-way ANOVA (**B**, **C**, and **D**) or Student’s t test (**G**, **H**, **I**, and **J**)

Given its overexpression in cancer cells, we reasoned that CALB2 might also function in cancer cells. We detected the expression of CALB2 in various human PDAC cell lines. Compared to the human pancreatic normal ductal epithelial (HPNE) and primary PDAC cell lines, CALB2 expression tended to be higher in the metastatic PDAC cell lines, such as AsPC-1 derived from ascites and CFPAC-1 derived from liver metastasis (Fig. 4A, S6A). To investigate the biological functions of CALB2 in PDAC cells, we constructed AsPC-1 cells with stable knockdown (KD) of CALB2 and CFPAC-1 cells with stable knockout (KO) of CALB2. In line with previous reports [34], the migratory ability of metastatic PDAC cells was greatly impaired after CALB2 KD or KO (Fig. 4B). Furthermore, CALB2 KD or KO notably inhibited PDAC cell proliferation (Fig. 4C) and potentiated their sensitivity to gemcitabine (Fig. 4D), which was consistent with our previous findings (Fig. 3I-J). To gain a better understanding of the effects of CALB2 in human PDAC tissues, the weighted correlation network analysis (WGCNA) was preformed to measure the correlations between gene modules and CALB2 expression using PDAC datasets from the CPTAC and TCGA project (Fig. S6B-C), and subsequently the gene modules with the highest correlation in each dataset were extracted to make an intersection (Fig. S6D). The enrichment analysis showed that CALB2 correlated with EMT, epithelial cell proliferation and migration (Fig. S6E), supporting the phenotypic experimental results in PDAC cells (Fig. 4B-C). Moreover, CALB2 is included in the EMTome database (www.EMTome.org) as an EMT-related gene [35]. RNA-seq analysis of CALB2-KD or control AsPC-1 revealed that CALB2 KD resulted in significant downregulation of pathways known to be involved in metastasis formation, such as EMT, focal adhesion, and hypoxia gene signatures [30](Fig. 4E, Supplementary Table S8-9). Additionally, the downregulation of N-cadherin and the upregulation of E-cadherin proteins were observed in CALB2 KD or KO PDAC cell lines (Fig. 4F), suggesting that downregulation of CALB2 impairs the EMT program in PDAC.

Given that PDAC cells utilize an EMT program during metastatic dissemination [36], and the proportion of CALB2^+^ cancer cells was significantly higher in the tumor with metastasis in our cohort (Fig. 1G), we preliminarily assumed that CALB2 facilitated PDAC metastasis in vivo. To test this hypothesis, PDAC liver metastasis model was established in NPG mice by splenic injection with CALB2-KO or control CFPAC-1 cells. Mice with CALB2-KO tumors had fewer liver metastases compared to the control group (Fig. 4G-H), indicating that CALB2 KO effectively blocked PDAC liver metastasis. Liver metastatic nodules were then isolated from each group to extract tissue RNA. RT-qPCR analysis revealed that the expressions of epithelial genes were significantly downregulated in the CALB2-KO group compared to the control group (Fig. 4I), while mesenchymal genes showed no significant differences between the two groups (Fig. 4J). This result suggested that downregulation of CALB2 inhibits epithelialization of tumor cells in liver metastases, which is thought to be essential for successful metastatic outgrowth in PDAC [37, 38]. Altogether, our results indicated that CALB2 is required for both EMT-involved metastasis and metastatic outgrowth of PDAC.

### CALB2 promotes PDAC metastasis through Ca^2+^-CXCL14 inflammatory axis

**Fig. 5:**
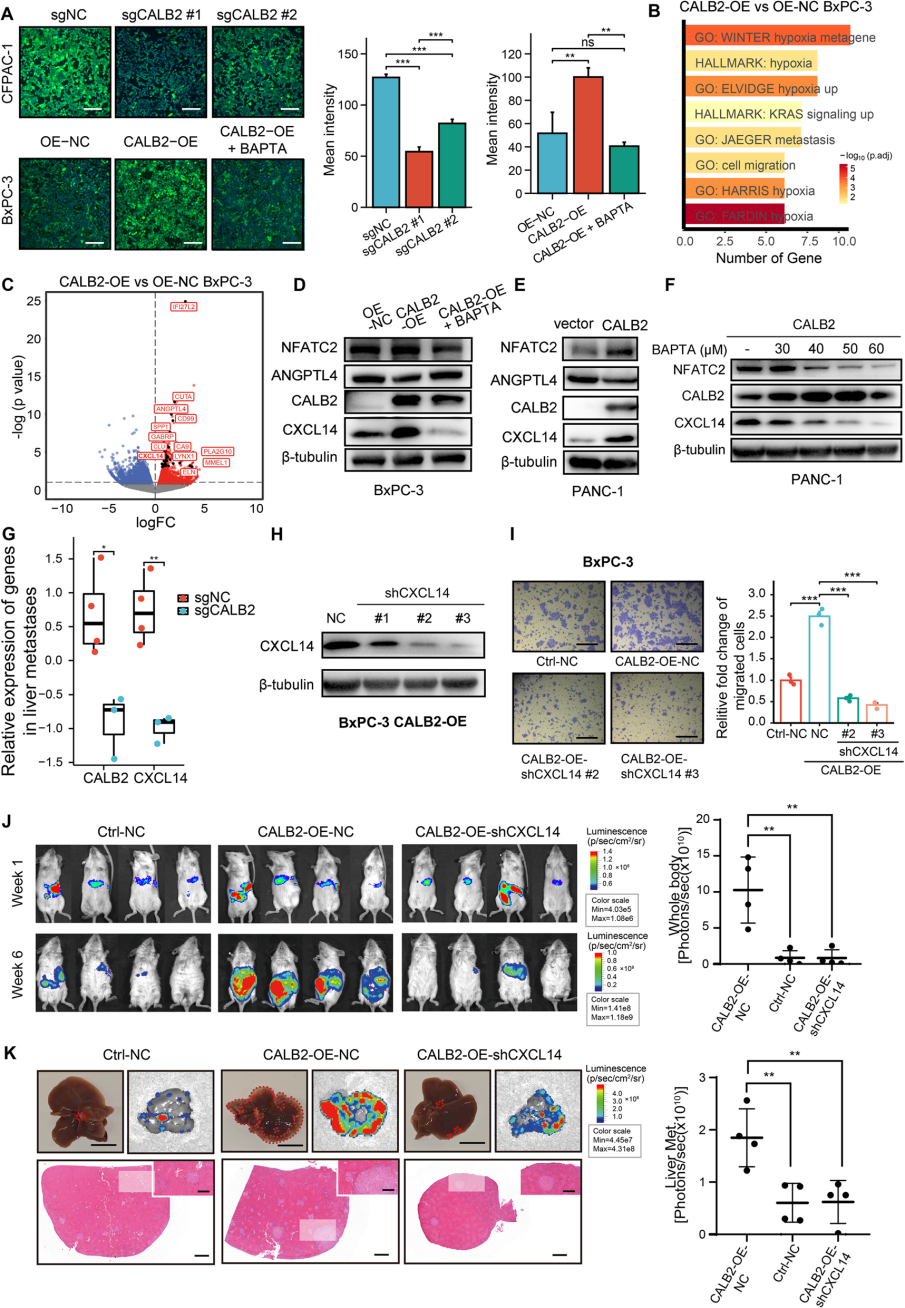
CALB2 promotes PDAC metastasis through Ca^2+^-CXCL14 inflammatory axis. (**A**) Representative Ca^2+^ fluorescence images (left) and quantification of intensity (right) in CALB2 knockdown (KO) CFPAC-1 cells, or stable CALB2-overexpressing (CALB2-OE) BxPC-3 cells with or without 20 μM BAPTA treatment for 48 h. (**B**) Significantly upregulated pathways (FDR < 0.05 and logFC > 0.5) identified by DAVID analysis of stable CALB2-OE BxPC-3 cells (*n* = 3) compared to stable OE control (OE-NC) BxPC-3 cells (*n* = 3). (**C**) Volcano plots partially showing significantly upregulated genes encoding secreted protein (*P* < 0.05 and logFC > 1) in CALB2-OE BxPC-3 cells compared to control BxPC-3 cells. (**D**) CALB2-OE BxPC-3 cells were treated with or without 20 μM BAPTA for 48 h, followed by western blotting. (**E**) PANC-1 cells were transfected with CALB2 or control vector, followed by western blotting to identify candidate downstream molecules of CALB2. (**F**) CALB2-transfected PANC-1 cells were treated with increased concentrations of BAPTA for 48 h, followed by western blotting to identify candidate downstream molecules regulated by CALB2-Ca^2+^ axis. (**G**) Relative expression of CXCL14 in liver metastases from stable CALB2-KO (*n* = 3) and control (*n* = 4) CFPAC-1 cells using RT-qPCR (2^−Δt^). (**H**) CALB2-OE BxPC-3 cells were subjected to stable CXCL14 KD (shCXCL14), followed by western blotting to confirm knockdown efficiency. (**I**) The effect of CXCL14 KD on the migration ability of CALB2-OE BxPC-3 cells was determined by Transwell assays. (**J**) IVIS living imaging and quantification of mice with splenic injection of shCXCL14 or control CALB2-OE stable BxPC-3 cells (*n* = 4 per group) at week 1 and week 6. (**K**) Representative ex vivo liver imaging and quantification, bright-field images of liver tissues, as well as H&E staining of metastatic nodules in mice from **J**. Scale bars, 200 μm (**A** and **I**), 1 cm (**K** top), 500 μm (**K** middle), 1000 μm (**K** bottom). Error bars, mean ± SD; *p < 0.05, **p < 0.01, ***p < 0.001, ****p < 0.0001; ns, not significant; by one-way ANOVA (**A**, **I**, **J**, and **K**) or Student’s t test (**G**)

CALB2 is a member of the EF-hand Ca^2+^-binding protein family mainly located in the cytosol, where it acts as a buffer for intracellular calcium ions [39]. Cellular Ca^2+^ homeostasis is crucial for the development of the metastatic cell phenotype and tumor cell migration [40]. Thus, we utilized a Ca^2+^ fluorescence probe to examine the effect of CALB2 on intracellular Ca^2+^ concentration. The Ca^2+^ fluorescence intensity was significantly decreased in CALB2 KO cells, whereas it was significantly increased in stable CALB2-overexpressing (CALB2-OE) BxPC-3 cells, which was subsequently reversed by the intracellular Ca^2+^ chelator BAPTA (MCE, #HY-100545) (Fig. 5A). To further investigate the potential mechanism by which CALB2 promotes PDAC metastasis, CALB2-OE BxPC-3 cells were sorted for RNA-seq. Consistent with the RNA analysis results of CALB2-KD AsPC-1, pathways known to be involved in metastasis formation, such as hypoxia and cell migration gene signatures, were significantly upregulated in CALB2-OE BxPC-3 compared to the control (Fig. 5B, Supplementary Table S10-11). Given the positive correlation between CALB2 and inflammatory response (Fig. S4B, S6E), we focused on CALB2-induced production of inflammatory cytokines, which might be responsible for PDAC metastasis. Significantly upregulated genes encoding secreted protein were identified in CALB2-OE BxPC-3 (Fig. 5C, Supplementary Table S12). We hypothesized that these elevated secretory factors relied on the function of CALB2 to buffer Ca^2+^ and thus screened them for downstream molecules by treating CALB2-OE BxPC-3 cells with BAPTA. The RNA levels of ANGPTL4 and CXCL14 increased when CALB2 was stably overexpressed but decreased after BAPTA treatment, indicating that they might be regulated by CALB2-Ca^2+^ axis (Fig. S7A). However, the alteration of protein levels in CALB2-OE BxPC-3 with BAPTA treatment suggested CXCL14 is more likely to be the candidate downstream molecule for CALB2-Ca^2+^ axis (Fig. 5D). To verify this hypothesis, PANC-1 cells were transfected with CALB2 vector and then treated with various concentrations of BAPTA. CXCL14 was also notably increased in CALB2-OE PANC-1 cells (Fig. 5E) but decreased when treated with high concentration of BAPTA (Fig. 5F). Additionally, CXCL14 was significantly downregulated in both CALB2-KO liver metastases in vivo (Fig. 5G) and CALB2-KO CFPAC-1 cells in vitro (Fig. 4F). In human PDAC tissues, CALB2 was positively correlated with CXCL14 expression (Fig. S3E). Both the RNA and protein abundance of CXCL14 was significantly elevated in tumor tissues compared to normal adjacent tissues (Fig. S7B-C). Similar to CALB2, EMT, hypoxia, and inflammatory response pathways were also significantly enriched in tumor tissues with high CXCL14 expression (Fig. S7D-E). Altogether, these results indicated that CXCL14 might serve as a downstream gene for CALB2.

To further investigate the effect of the CALB2-CXCL14 axis on PDAC metastasis, we subsequently established CALB2-OE-NC, and CALB2-OE-shCXCL14 BxPC-3 stable cell lines (Fig. 5H). The in vitro migratory capability was significantly increased in the CALB2-OE-NC group compared with the Ctrl-NC group, while this effect was notably reversed by CXCL14 KD (Fig. 5I). Finally, the three BxPC-3 cell lines (Ctrl-NC, CALB2-OE-NC, and CALB2-OE-shCXCL14) were injected into the spleen of NPG mice (Fig. 5J-K). Larger liver metastasis area was observed in the CALB2-OE-NC group compared to the Ctrl-NC group. CXCL14 KD in CALB2-OE cells significantly reduced the liver metastasis induced by CALB2 overexpression, compared with CALB2-OE-NC cells. Overall, our results demonstrated that CALB2 facilitates PDAC metastasis, presumably by Ca^2+^-CXCL14 inflammatory axis.

### CALB2 promotes immunosuppression and metastasis in KPC organoid allograft mice

**Fig. 6:**
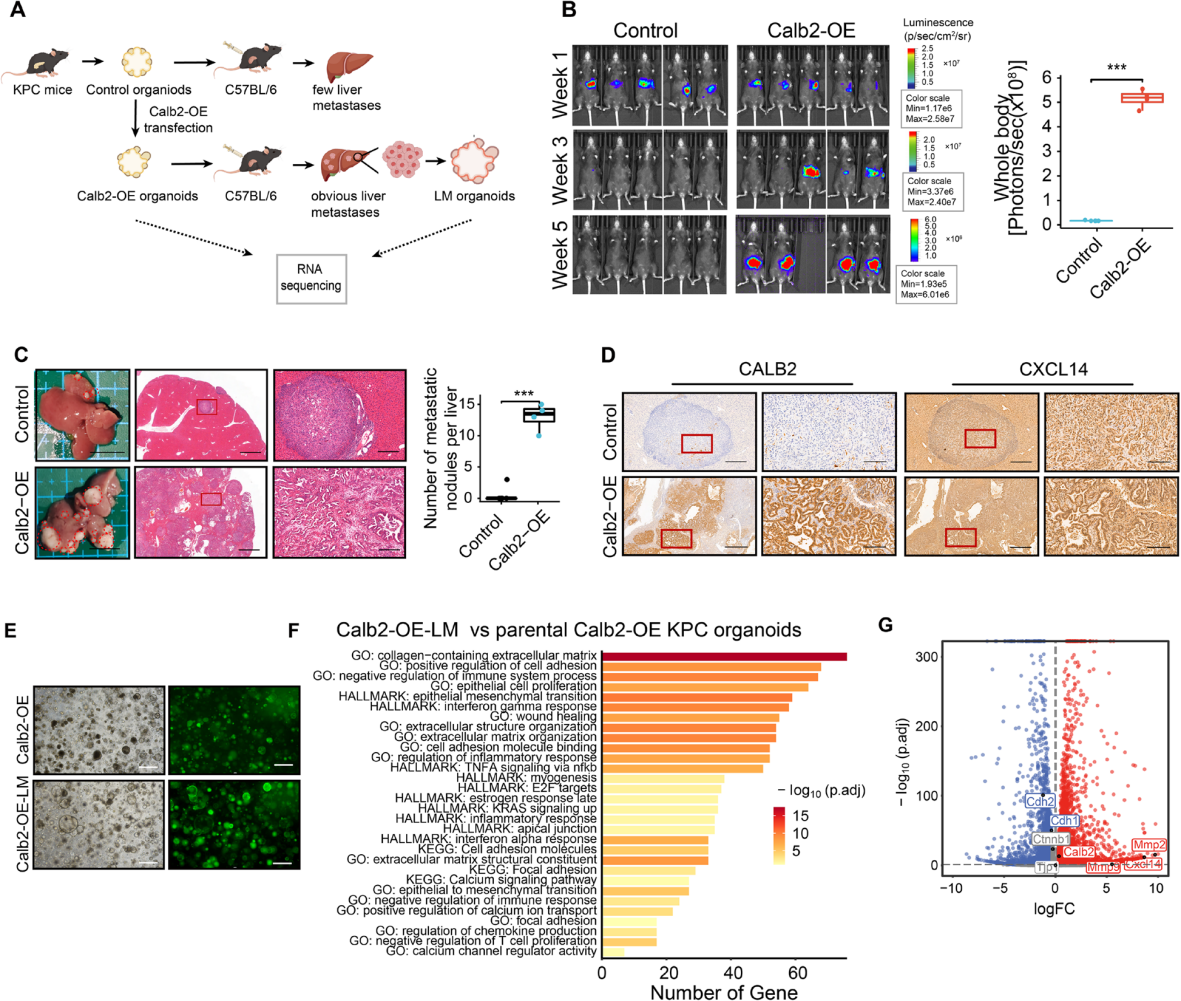
CALB2 promotes immunosuppression and metastasis in KPC organoid allograft mice. (**A**) Schematic diagram depicting KPC organoid xenografts in the C57BL/6 mouse model. KPC organoids were stably transfected with Calb2-OE or control lentivirus and subsequently injected into the spleen of C57BL/6 mice to compare their ability to liver metastasis. (**B**) IVIS living imaging of the whole body (left) at week 1, 3, and 5, and quantification of the signals at week 5 (*n* = 5 each group; right). (**C)** Representative bright-field images and H&E staining images for metastatic lesions (left) and the quantification of metastatic nodules (right). Scale bars: 1 cm (left), 600 μm (middle), 200 μm (right). (**D**) Metastatic liver sections from **C** stained with IHC for CALB2 and CXCL14. Scale bars, 500 μm (left), 200 μm (right). (**E**) Representative microscopy images of bright field and green fluorescence of the indicated KPC organoids following single-cell passage for 5 days. Scale bars, 500 μm. (**F**) Significantly upregulated pathways (FDR < 0.05 and logFC > 1) identified by DAVID analysis of Calb2-OE liver metastasis (LM) KPC organoids (*n* = 3) compared to Calb2-OE KPC organoids (*n* = 3). (**G**) Volcano plots partially showing differential expression of Calb2, Cxcl14, and EMT-related genes in Calb2-OE-LM KPC organoids compared to Calb2-OE KPC organoids. Red, significant upregulated genes; blue, significantly downregulated genes; grey, non-significantly changed genes. Error bars, mean ± SD; *p < 0.05, **p < 0.01, ***p < 0.001, ****p < 0.0001; ns, not significant; by Student’s t test (**B** and **C**)

Given CALB2 is negatively correlated with the immunosuppressive TME (Fig. S1C, S3C-E), a critical question is whether CALB2 also promotes PDAC metastasis under normal immune surveillance. Immunodeficient mice are commonly used in most research on tumor metastasis since immunosurveillance inhibits the survival and metastasis of tumor grafts in vivo, however, this model overlooks the influence of adaptive immunity on cancer metastasis. Thus, we injected organoids isolated from the primary PDAC of the LSL-K-Ras^G12D/+^; LSL-p53^R172H/+^; Pdx1-Cre (KPC) genetically engineered mouse model into syngeneic immunocompetent mice to establish KPC organoid allograft mice (Fig. 6A). The KPC organoids with stable Calb2 overexpression (Calb2-OE) or the control were utilized to generate the liver metastasis model by splenic injection. After 3 weeks post-injection, most of the allografts nearly disappeared in the control group, whereas most of mice in the Calb2-OE group still had tumors (Fig. 6B). After 5 weeks, the Calb2-OE group showed significantly more and larger liver metastases, with some mice even succumbing to metastasis, in contrast to the control group, where the mice either showed no obvious liver metastases or only had several small ones (Fig. 6C). These results suggested that CALB2 plays an immunosuppressive role in the survival and metastasis of PDAC organoid allografts. Moreover, liver metastases from the Calb2-OE group exhibited higher CXCL14 expression compared to the control group (Fig. 6D). To examine the functional heterogeneity following liver metastasis, organoids were established from the liver metastases of the Calb2-OE group (Calb2-OE-LM) and subsequently harvested for RNA-seq analysis. Calb2-OE-LM organoids proliferated better compared to their parental Calb2-OE organoids in vitro (Fig. 6E). In line with our previous findings, pathways of inflammatory response, epithelial cell proliferation, positive regulation of calcium ion, and negative regulation of immune, as well as pathways known to be involved in metastasis formation, such as EMT, cell adhesion, and wound healing, were significantly upregulated in Calb2-OE-LM organoids compared to Calb2-OE organoids (Fig. 6F, Supplementary Table S14). Notably, Calb2 and Cxcl14 were significantly upregulated in Calb2-OE-LM organoids (Fig. 6G, Supplementary Table S13). These findings indicated the immunosuppressive role of CALB2 in PDAC metastasis.

### Combination of CXCL14 neutralizing antibody with gemcitabine effectively hinders CALB2-mediated metastasis and improves survival outcome

**Fig. 7:**
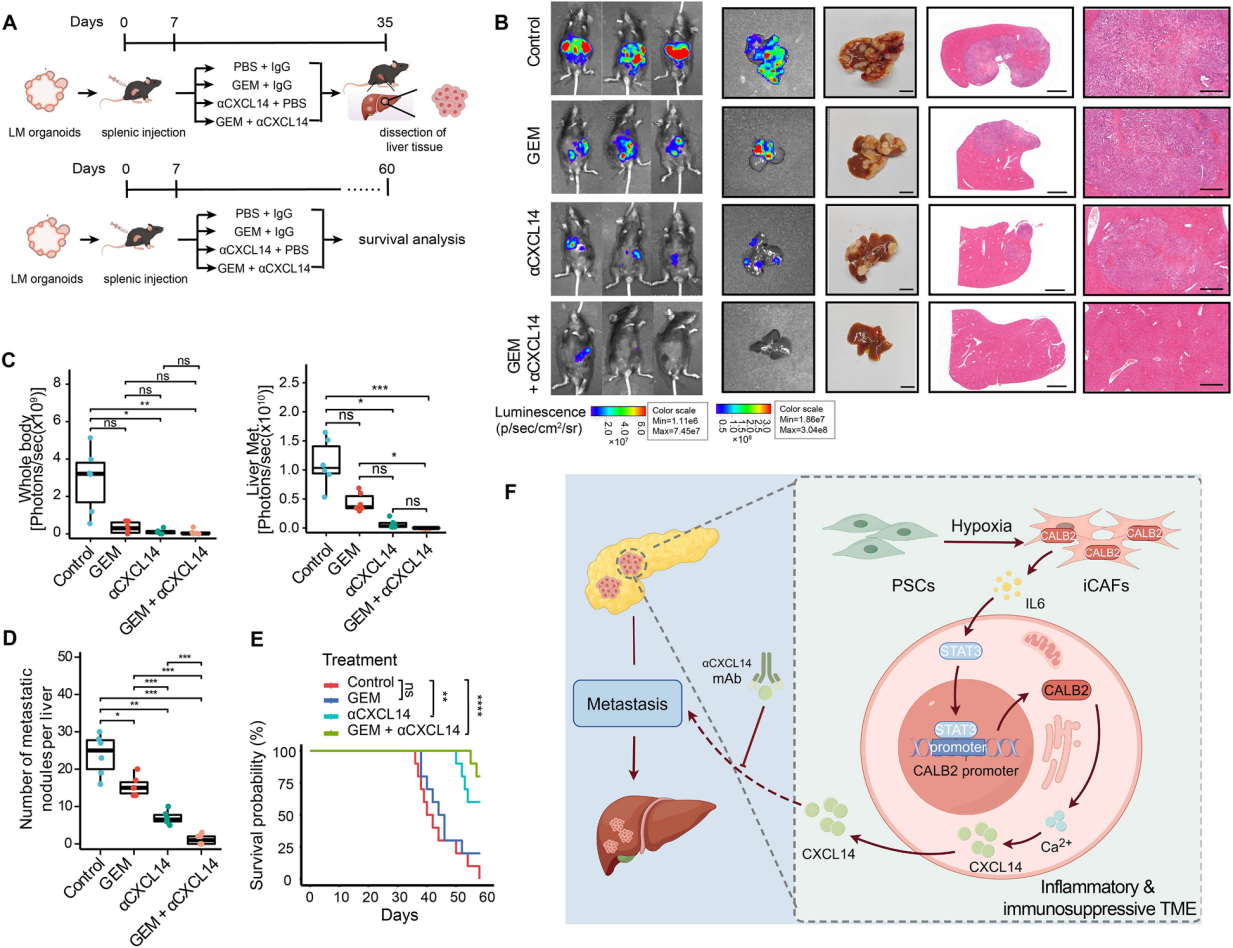
Combination of CXCL14 neutralizing antibody with gemcitabine effectively hinders CALB2-mediated metastasis and improves survival outcome. (**A**) Liver metastases from the Calb2-OE tumor were isolated to establish liver metastasis (LM) organoids. After one week of splenic injection of LM organoids into the C57BL/6 mice, isotype IgG, αCXCL14 (1 mg/kg, intravenously), gemcitabine (25 mg/kg, intraperitoneally) or combined chemotherapy (αCXCL14 + gemcitabine) were injected twice per week for 4 consecutive weeks (top). Animal survival was monitored up to 60 days after injection (bottom). (**B**) Representative IVIS bioluminescence images, bright-field images, and H&E staining for metastatic lesions after 5 weeks of splenic injection of Calb2-OE-LM KPC organoids. Scale bars: 1 cm (left), 2000 μm (middle), 500 μm (right). (**C-D**) Quantification of living imaging, ex vivo liver imaging (**C**) and metastatic nodules (**D**) from **B** (*n* = 6 per group). (**E**) Kaplan-Meier survival curves for mice with KPC liver metastasis-derived organoid xenografts treated with the indicated regimen (*n* = 10 per group). (**F**) Schematic diagram illustrating the inflammatory reprogramming mechanism by which CALB2 promotes liver metastasis of PDAC. Error bars, mean ± SD; *p < 0.05, **p < 0.01, ***p < 0.001, ****p < 0.0001; ns, not significant; by one-way ANOVA (**C-D**) or log-rank test (**E**)

To further determine whether targeting CALB2-CXCL14 inflammatory axis can alleviate PDAC metastasis in immunocompetent conditions, we evaluated the therapeutic effect of αCXCL14 monoclonal antibody (mAb) and combination GEM chemotherapy on the liver metastasis of Calb2-OE-LM organoid allografts (Fig. 7A). Calb2-OE-LM organoid allografts were selected as experimental models because they had a more stable and higher liver metastasis rate compared to Calb2-OE organoid allografts. After 5 weeks post-injection, treatment with GEM had limited efficacy on liver metastasis, while intravenous administration of αCXCL14 mAb as monotherapy effectively suppressed PDAC metastasis, and combination therapy with GEM exhibited a more potent inhibitory effect on liver metastasis formation (Fig. 6B-D). These results suggested that αCXCL14 mAb not only blocks PDAC metastasis, but also potentiates the sensitivity of metastatic tumor to GEM. Furthermore, the administration of αCXCL14 mAb as monotherapy or in combination with GEM chemotherapy notably extended the survival of KPC organoid allograft mice (Fig. 6E). Therefore, blockade of CALB2-CXCL14 inflammatory axis may serve as an effective approach to inhibit PDAC metastasis.

Taken together, the above experimental findings indicate that CALB2 collaborates with hypoxic TME to activate an inflammatory CAF phenotype. CALB2 is upregulated in cancer cells by IL6-STAT3 inflammatory signaling-mediated direct transcription. The CALB2-Ca^2+^-CXCL14 inflammatory axis confers highly metastatic capability upon cancer cells and further facilitates an inflammatory and immunosuppressive TME in PDAC. Inflammation-targeted therapy with αCXCL14 monoclonal antibody emerges as a promising strategy to suppress CALB2-mediated metastasis of PDAC (Fig. 7F).

## Discussion

PDAC exhibits uniquely resistant to standard chemotherapies and is prone to early metastasis. Chronic, dysregulated, persistent and unresolved inflammation provides a preferred tumor microenvironment for tumorigenesis, development, and metastasis. A better understanding of the key regulators that maintain inflammatory tumor microenvironment and the development of predictive biomarkers to identify patients who are most likely to benefit from specific inflammatory-targeted therapies are crucial for advancing personalized cancer treatment. In this study, we highlight the central role of CALB2 in driving metastatic progression through inflammatory reprogramming and uncover a therapeutically accessible target, CXCL14, as the central mediator of metastasis downstream of CALB2 overexpression.

CALB2, a 29-kDa calcium-binding protein from the EF-hand protein family with a high affinity to calcium ions, is primarily considered as a cytosolic calcium buffering protein and also functions as a calcium sensor protein [41, 42]. Its roles in neurons are well recognized [43–45]. However, its roles in CAFs have not been explored. Proteomic analysis of laser-capture microdissected PDAC samples revealed that CALB2 serves as a compartment-specific poor prognostic marker for the tumor area [25, 46]. Herein, we found that CALB2 is specifically expressed in both CAFs and cancer cells within the tumor tissue using human PDAC scRNA-seq analysis. Our larger human PDAC tissue microarray cohort confirmed that CALB2 is not only a tumor-specific but also a CAF-associated poor prognostic marker, indicating that CALB2 might also exert biological effects on CAFs. Both iCAFs and myCAFs can be generated from pancreatic stellate cells (PSCs), a major source of CAFs in PDAC [20]. Siel Olbrecht et al. identified a fibroblast subtype derived from the mesothelium in high-grade serous tubo-ovarian cancer, named FB_CALB2, which expresses high levels of CALB2, pro-inflammatory cytokines (IL6 and IL18) and IL6-associated genes promoting fibrosis (COL8A1, CXCL16) [24]. Consistently, we found significantly elevated CAF markers, IL6 production and cell proliferation in PSCs stably overexpressing CALB2, indicating that CALB2 promotes the transition of PSCs to an iCAF phenotype. Increased demand from cancer cells and impaired vascularization creates a hypoxic TME in PDAC, which causally promotes acquisition of an iCAF state [31, 47, 48]. In line with this, we demonstrated a significant upregulation of hypoxia and inflammatory response gene signatures in CALB2^+^ CAFs compared to PSCs and CALB2^−^ CAFs. Furthermore, CALB2 and IL6 expressions are increased in the process that PSCs are activated to iCAFs under hypoxic conditions. iCAFs stimulate paracrine signaling through elevated expression of cytokines and chemokines to promote tumor growth, cell survival, and metastasis [20]. We found that transwell co-culture with CALB2^+^ CAFs promotes human PDAC organoid growth and cell migration in vitro. Pancreatic transplantation of organoids in NPG mice (PDOX model) further demonstrated that CALB2^+^ CAFs promote tumor growth in vivo. Therefore, this study demonstrated that CALB2 can collaborate with hypoxia to promote iCAF activation and CALB2-activated CAFs play a tumor-promoting role in PDAC progression, which enhances our understanding of the molecular and functional heterogeneity of CAFs.

Additionally, we revealed an inflammation-dependent cyclical mechanism by which CALB2-activated CAFs in turn upregulate CALB2 expression in cancer cells. iCAFs are the significant source of IL6 in the PDAC TME, with the ability to activate the STAT3 pathway in cancer cells [20, 49]. Consistently, we found that CALB2-activated CAFs secrete IL6 to stimulate STAT3 signaling in PDAC cells. The activated STAT3 directly binds to the gene promoter of CALB2 and regulates CALB2 transcription, indicating that IL6-STAT3 inflammatory signaling is responsible for CALB2 upregulation in cancer cells.

CALB2 is generally considered an oncogenic gene and a poor prognostic factor in current cancer research. Tissue CALB2 is clinically used to distinguish CALB2-positive epithelioid and mixed-type (biphasic) malignant mesothelioma (MM) from adenocarcinoma, while serum CALB2 could potentially serve as a predictive biomarker for poor survival and outcomes of cisplatin-based chemotherapy in MM [50]. Moreover, CALB2 is not expressed in normal colon cells, but it is expressed in most poorly differentiated colon cancers [51], for example, high levels of expression of CALB2 are observed in medullary carcinoma of the colon with strong focal IHC staining, emphasizing its utility as a biomarker for colon cancer [52]. In addition to serving as a biomarker for malignancy, CALB2 also plays a vital role in tumor metastasis. In ovarian high-grade serous carcinoma, which is characterized by early and extensive peritoneal dissemination, CALB2 is upregulated in tumor cells of the metastases, particularly at the invasive tumor edges, and contributes to cancer cell adhesion to the extracellular matrix (ECM) of peritoneal organs [53]. CALB2 has been proposed to promote hepatocellular carcinoma metastasis via the TRPV2-Ca^2+^-ERK1/2 signaling pathway [39]. In PDAC, CALB2 overexpression counteracts CSTF2T knockdown-mediated suppression of growth and metastasis in vivo [34]. Nevertheless, the potential mechanism and therapeutic strategy for CALB2-driven PDAC metastasis remain elusive. In this study, we confirmed that CALB2 is significantly higher in tumor with metastasis using human PDAC tissue microarray, and it promotes malignant phenotypes, especially metastatic growth through loss of function in human metastatic PDAC cells or gain of function in primary PDAC cells. Furthermore, we found that CALB2 also promotes liver metastasis in immunocompetent KPC organoid allograft mice, suggesting the potential immunosuppressive role of CALB2 in PDAC metastasis. Mechanistically, we demonstrated that CALB2 activates Ca^2+^-CXCL14 inflammatory axis, indicating that CALB2 also elicits inflammation in epithelial cells to enhance metastatic ability. Thus, our study elucidates an inflammatory reprogramming mediated by CALB2 in the TME to sustain the malignant potential of PDAC.

The role of CXCL14 in cancer development is complex and contradictory. Both oncogenic and tumor-suppressive functions of CXCL14 have been reported among various cancers [54–57]. CXCL14 is increased in the invasive front of PDAC specimens, and treatment with CXCL14 significantly increases the invasiveness of pancreatic cancer cells, suggesting that CXCL14 may function as an oncogene in PDAC [58]. Recent studies have shown that cancer cell-intrinsic CXCL14 also promotes EMT and distant metastasis in breast cancer [59], lung cancer [60], and osteosarcoma [61], apart from CAF-derived CXCL14 [62]. Consistently, our study demonstrated that genetic or pharmacological blockade of CXCL14 effectively inhibits CALB2-mediated PDAC metastasis in immunodeficient or immunocompetent models, indicating that CALB2-mediated intrinsic upregulation of CXCL14 in cancer cells promotes PDAC metastasis and CXCL14 might potentiate the immunosuppressive role of CALB2. Combination chemotherapy with αCXCL14 monoclonal antibody emerges as a promising strategy to suppress distant metastasis and improve survival outcomes in PDAC with CALB2 overexpression. Further work will be required to evaluate the potential direct immunomodulatory effects of CALB2-CXCL14 inflammatory axis and to fully understand how these processes cooperate to drive PDAC metastatic progression.

## Conclusions

In summary, our findings identify CALB2 as a key regulator of inflammatory reprogramming to drive PDAC metastasis. CALB2 collaborates with hypoxia to activate iCAFs, which secrete IL6 to upregulate CALB2 expression in cancer cells via STAT3-mediated direct transcription. CALB2-Ca^2+^-CXCL14 inflammatory axis confers highly metastatic and immunosuppressive ability on cancer cells. Our study also provides a theoretical basis for the clinical application of αCXCL14 monoclonal antibody combined with gemcitabine chemotherapy to inhibit distant metastasis in PDAC patients with CALB2 overexpression.

## Supplementary Information

Supplementary Material 1.

Supplementary Material 2.

Supplementary Material 3.

Supplementary Material 4.

## Data Availability

Expression profiles analyzed in this study were obtained from GEO database at GSE101448, GSE21501, GSE28735, GSE57495, GSE62165, GE62452, GSE71729, GSE78229, GSE79668, and GSE85916 or from ICGC database at PAAD_AU and PAAD_CA, or from ArrayExpress database at E-MTAB-6134. The clinical data and gene expression matrix of the TCGA pancreatic adenocarcinoma (PAAD) project were obtained from cBioPortal (https://www.cbioportal.org/). The mRNA expression values and protein abundance of CALB2 and CXCL14 in normal or pancreatic tumor tissues from the Clinical Proteomic Tumor Analysis Consortium (CPTAC) project were downloaded directly from cProSite (https://cprosite.ccr.cancer.gov/). The single-cell RNA sequencing data was obtained from the Genome Sequence Archive (SRA) in the National Center for Biotechnology Information (NCBI) database under the project PRJCA001063. The raw RNA sequencing data reported in this article have been deposited in the SRA under BioProject accession numbers PRJNA1132205, PRJNA1132214 and PRJNA1132248. All other raw data generated or analyzed in this study are available upon request from the corresponding author.

## Abbreviations

AJCC: American Joint Committee on Cancer
apCAFs: Antigen presenting CAFs
CAFs: Cancer-associated fibroblasts
CM: Conditioned medium
DEGs: Differentially expressed genes
DFS: Disease-free survival
DSS: Disease-specific survival
ECM: Extracellular matrix
EMT: Epithelial to mesenchymal transition
FAP: Fibroblast activation protein
GEM: Gemcitabine
GO: Gene Ontology
GSEA: Gene set enrichment analysis
GSVA: Gene set variation analyses
H&E: Hematoxylin and eosin
iCAFs: Inflammatory CAFs
IHC: Immunohistochemistry
KD: Knockdown
KO: Knockout
KPC: LSL-K-Ras^LSLG12D/ +^ ; LSL-p53^R172H/ +^ ; Pdx1-Cre
mAb: Monoclonal antibody
MM: Malignant mesothelioma
MSigDB: Molecular Signatures Database
myCAFs: myofibroblastic CAFs
NCBI: National Center for Biotechnology Information
OS: Overall survival
PDAC: Pancreatic ductal adenocarcinoma
PDOs: Patient-derived organoids
PDOX: Patient-derived organoid xenografts
PFS: Progression-free survival
PSCs: Pancreatic stellate cells
PUMCH: Peking Union Medical College Hospital
RFS: Recurrence free survival
rhIL6: Recombinant human IL6
RT: Room temperature
scRNA-seq: Single-cell RNA sequencing
TMA: Tissue microarray
TME: Tumor microenvironment
WGCNA: Weighted correlation network analysis
