# Supplementary material for: CALB2 drives pancreatic cancer metastasis through inflammatory reprogramming of the tumor microenvironment
Source: J Exp Clin Cancer Res. 2024 Oct 3;43:277. doi: 10.1186/s13046-024-03201-w (PMC11448066; doi:10.1186/s13046-024-03201-w)
Supplement: Supplementary file 1 — Supplementary Material 1. [file 13046_2024_3201_MOESM1_ESM.docx]

**Supplementary figures and figure legends**

**
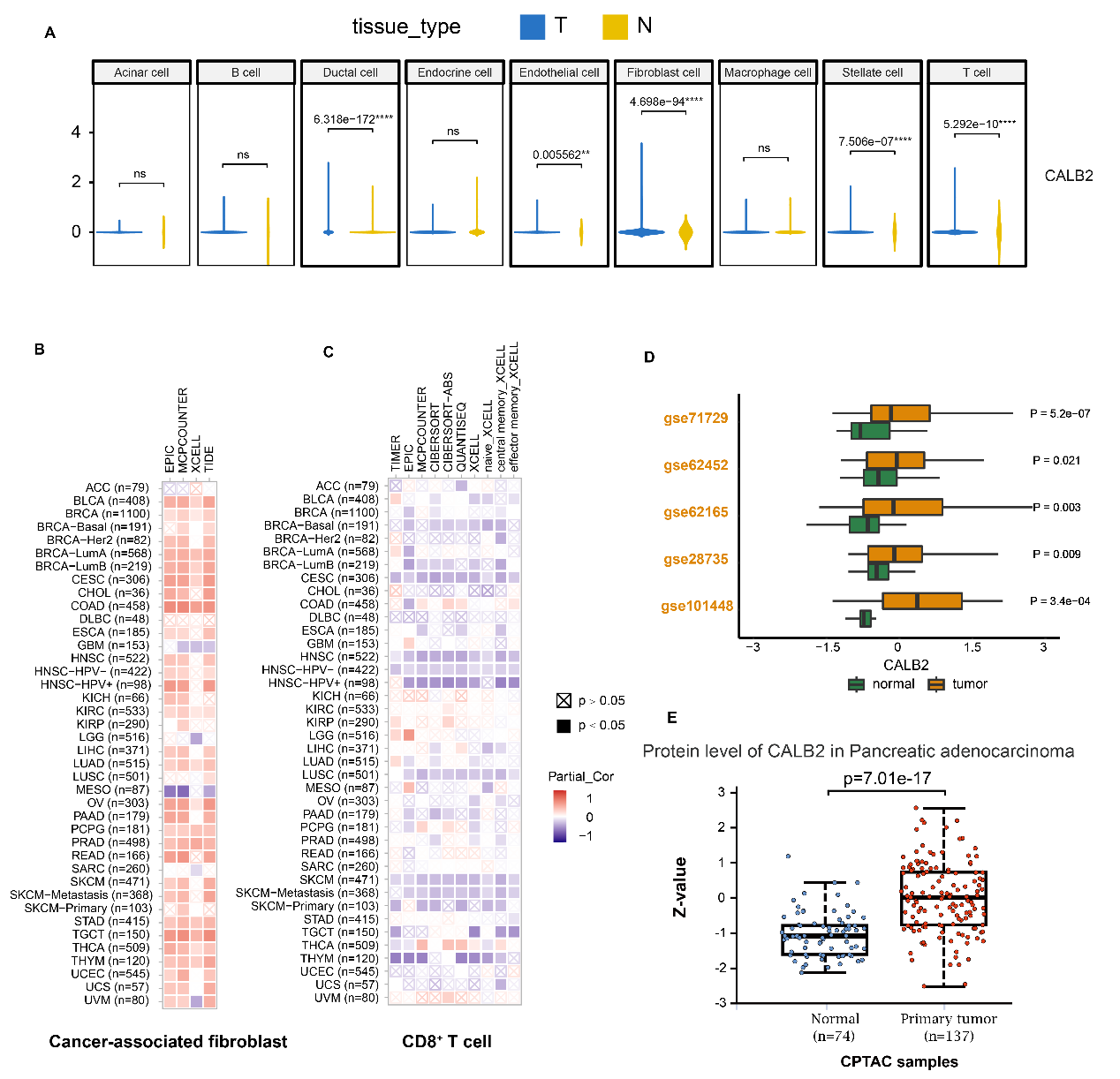
**

**Fig. S1. CALB2 RNA abundance is notably elevated in PDAC tumor tissues and correlates with high abundance of CAFs and low abundance of CD8^+^ T cells. (A)** Violin plots showing expression levels of CALB2 for each cell type in tumor (T) and adjacent normal (N) tissues using the PDAC scRNA-seq data. **(B-C)** Heatmap illustrating the correlations of CALB2 expression with the abundance of CAFs (**B**) and CD8^+^ T cells (**C**) across pan-cancer transcript data using different algorithms. **(D)** Comparison of CALB2 expression in tumor and adjacent normal tissues across multiple PDAC transcriptome datasets. **(E)** Comparison of the protein abundance of CALB2 in PDAC tumor and adjacent normal tissues using the Clinical Proteomic Tumor Analysis Consortium (CPTAC) data. Error bars, mean ± SD; *p < 0.05, **p < 0.01, ***p < 0.001, ****p < 0.0001; ns, not significant; by Student’s t test (**D**, **E**).

**
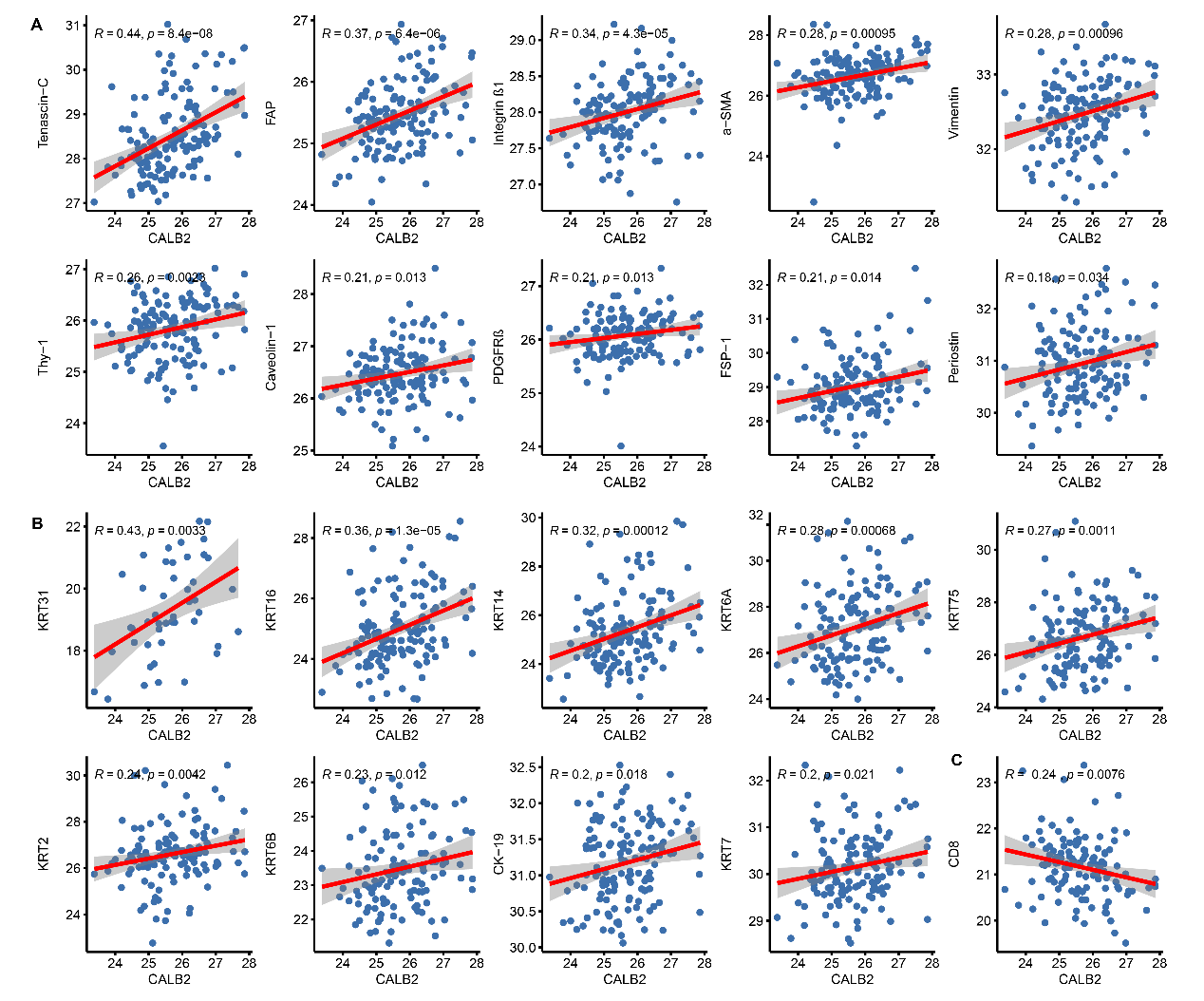
**

**Fig. S2. CALB2 protein level correlates with high abundance of CAF and epithelial markers and low abundance of CD8. (A-C)** Scatter plot demonstrating the correlations between CALB2 expression and classical markers of CAFs (**A**) and epithelial cells (**B**) as well as CD8^+^ T cells (**C**) using PDAC proteomic data from the CPTAC project.

**
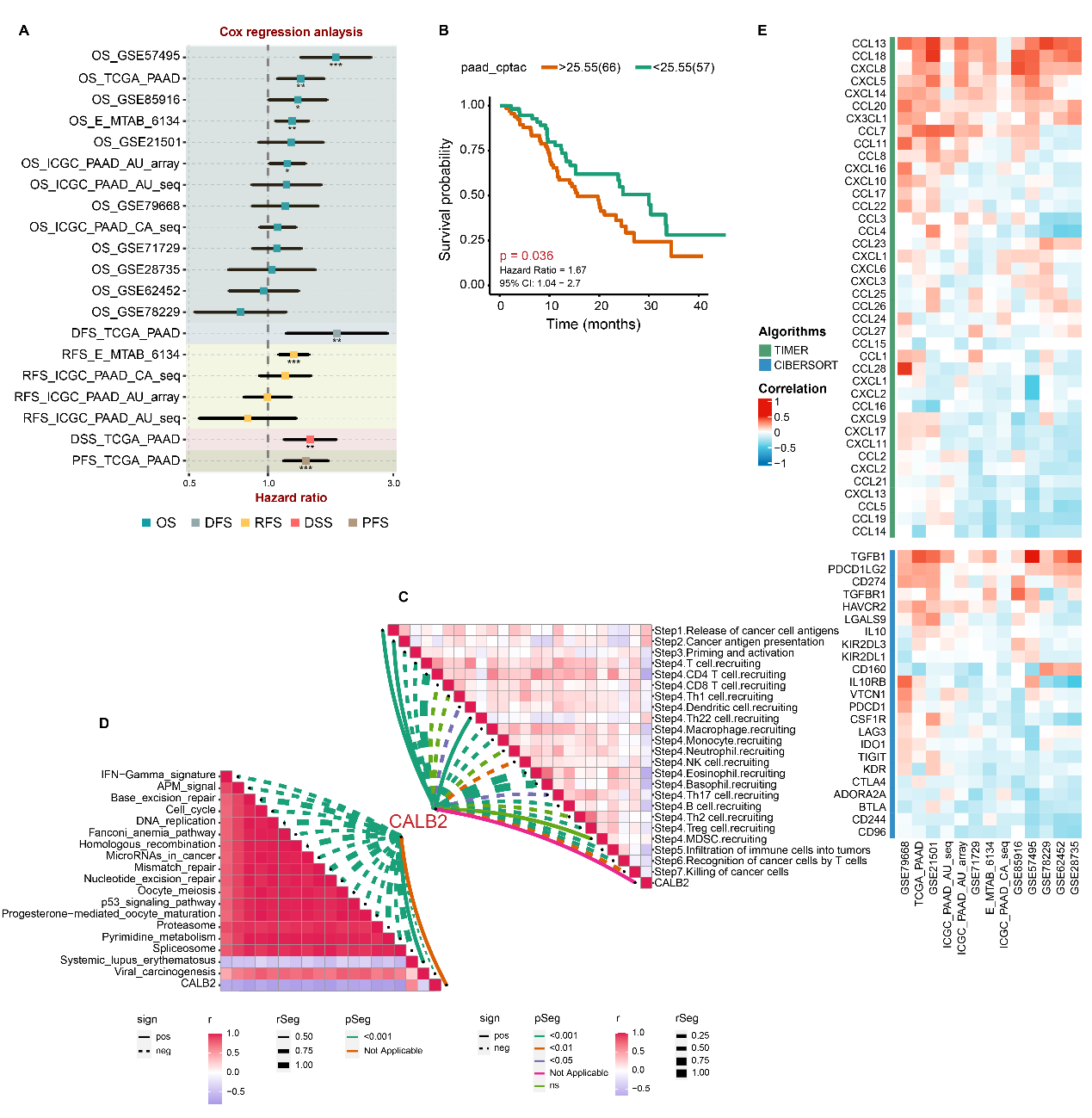
**

**Fig. S3. CALB2 correlates with an unfavorable patient prognosis and the immunosuppressive TME. (A)** Cox forest plot demonstrating Cox regression analysis of five survival variables in multiple PDAC datasets. **(B)** Kaplan-Meier curves with log-rank test based on CALB2 expression using PDAC proteomic data from the CPTAC project. **(C)** Correlations between CALB2 and the steps of the cancer immunity cycle. **(D)** Correlations between CALB2 and the enrichment scores of immunotherapy-predicted pathways. **(E)** Heatmaps illustrating the correlations of CALB2 with immunomodulators across multiple PDAC datasets.

**
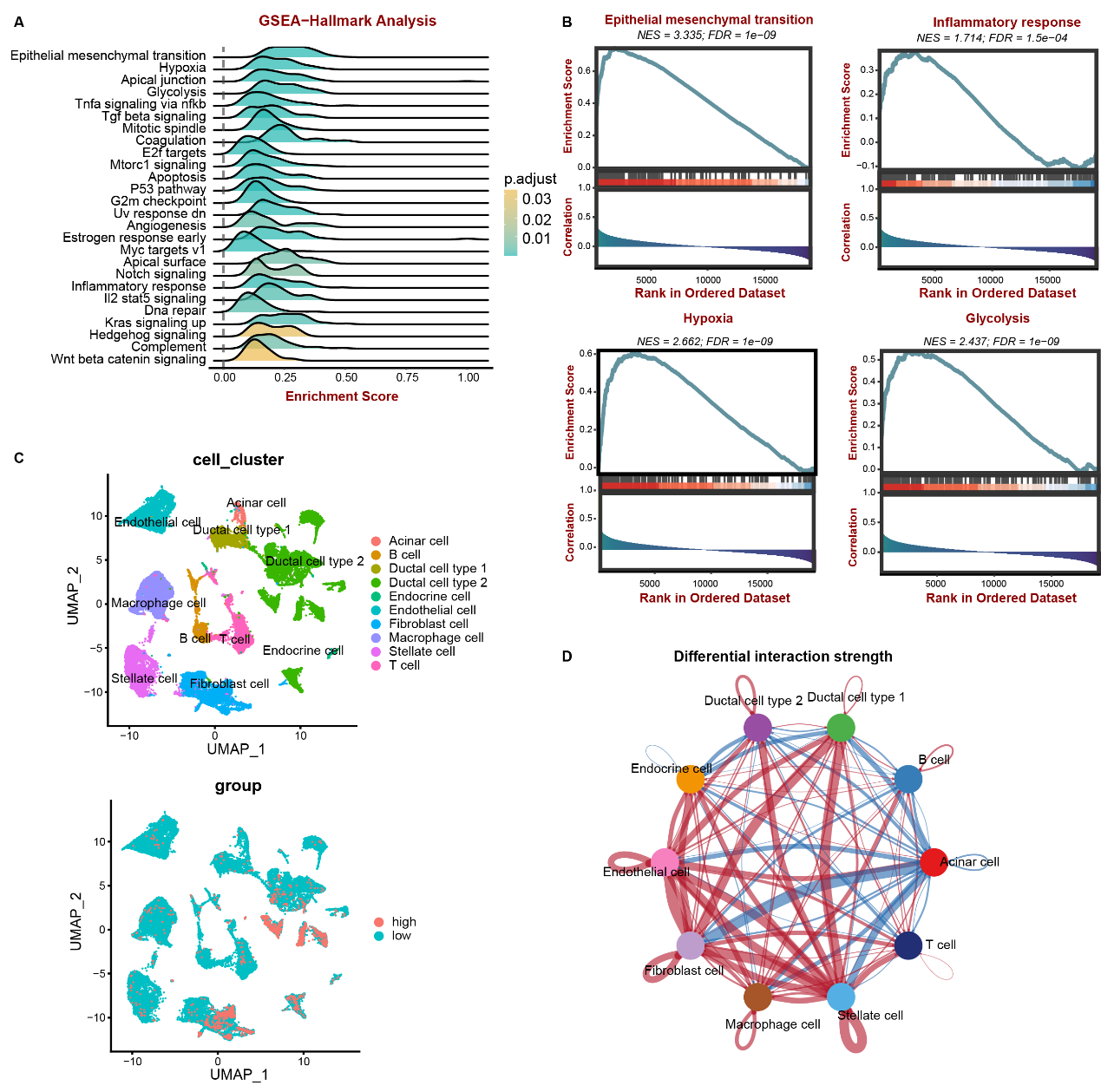
**

**Fig. S4. Hypoxia collaborates with CALB2 to promote the activation of CAFs and their crosstalk with neoplastic cells. (A-B)** Ridge-Plot (**A**) and GSEA-Plot (**B**) for the GSEA-Hallmark enrichment analysis. **(C)** UMAP plots of single-cell transcriptome data showing all cells were classified into two groups based on CALB2 expression. **(D)** Circle plot demonstrating differential cell-cell communication strength between CALB2-high and -low expression cells.

**
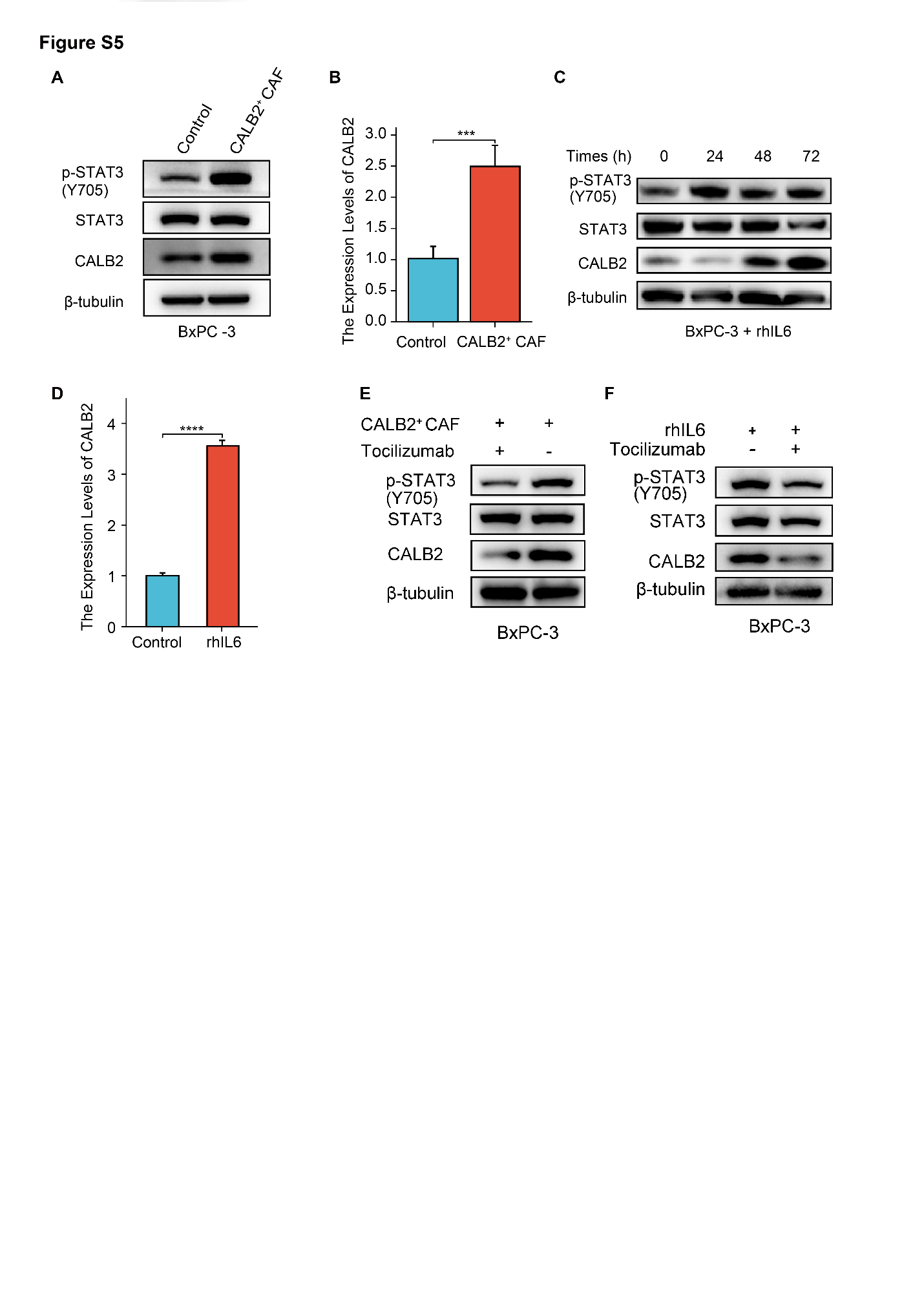
**

**Fig. S5. CALB2^+^ CAFs transcriptionally activates CALB2 expression in PDAC cells through IL6-STAT3 signaling pathway. (A-B)** BxPC-3 cells were co-cultured with CALB2^+^ CAFs or control CAFs for 72 h, followed by western blotting (**A**) and RT-qPCR analysis (**B**). **(C-D)** BxPC-3 cells were treated with 100 ng/mL rhIL6 over the time, followed by western blotting **(C)** and RT-qPCR analysis of 72 h treatment with rhIL6 **(D)**. **(E-F)** BxPC-3 cells were pre-treated with 2.5 μg/ml Tocilizumab (IL-6R neutralizing antibody) for 24h, then cultured with CALB2^+^ CAFs (**E**) or treated with 100 ng/mL rhIL6 (**F**) for 48 h, followed by western blotting. Error bars, mean ± SD; *p < 0.05, **p < 0.01, ***p < 0.001, ****p < 0.0001; ns, not significant; by Student’s t test (**B** and **D**).

**
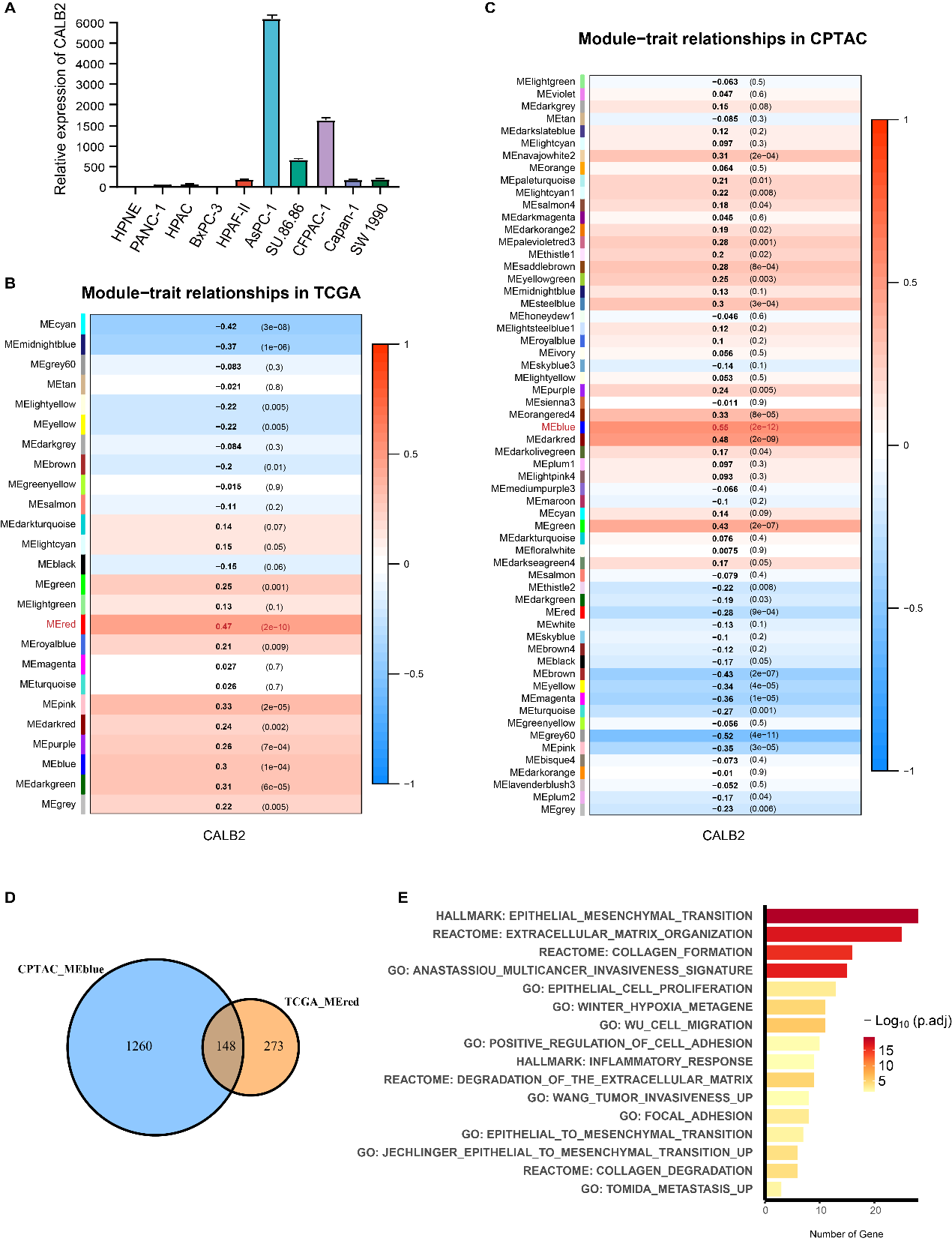
**

**Fig. S6. CALB2 promotes EMT of PDAC cells. (A)** RT-qPCR of CALB2 expression in human normal pancreatic ductal cells (hTERT-HPNE), and human primary and metastatic PDAC cell lines. **(B-C)** Correlation analysis between module eigengenes and CALB2 using PDAC transcriptome data from the TCGA **(B)** and CPTAC **(C)**. **(D)** Venn analysis of the gene modules with the highest correlation in TCGA and CPTAC. **(E)** Significantly enriched pathways (FDR < 0.05) identified by DAVID analysis of the intersected genes in **D**. Error bars, mean ± SD.

**
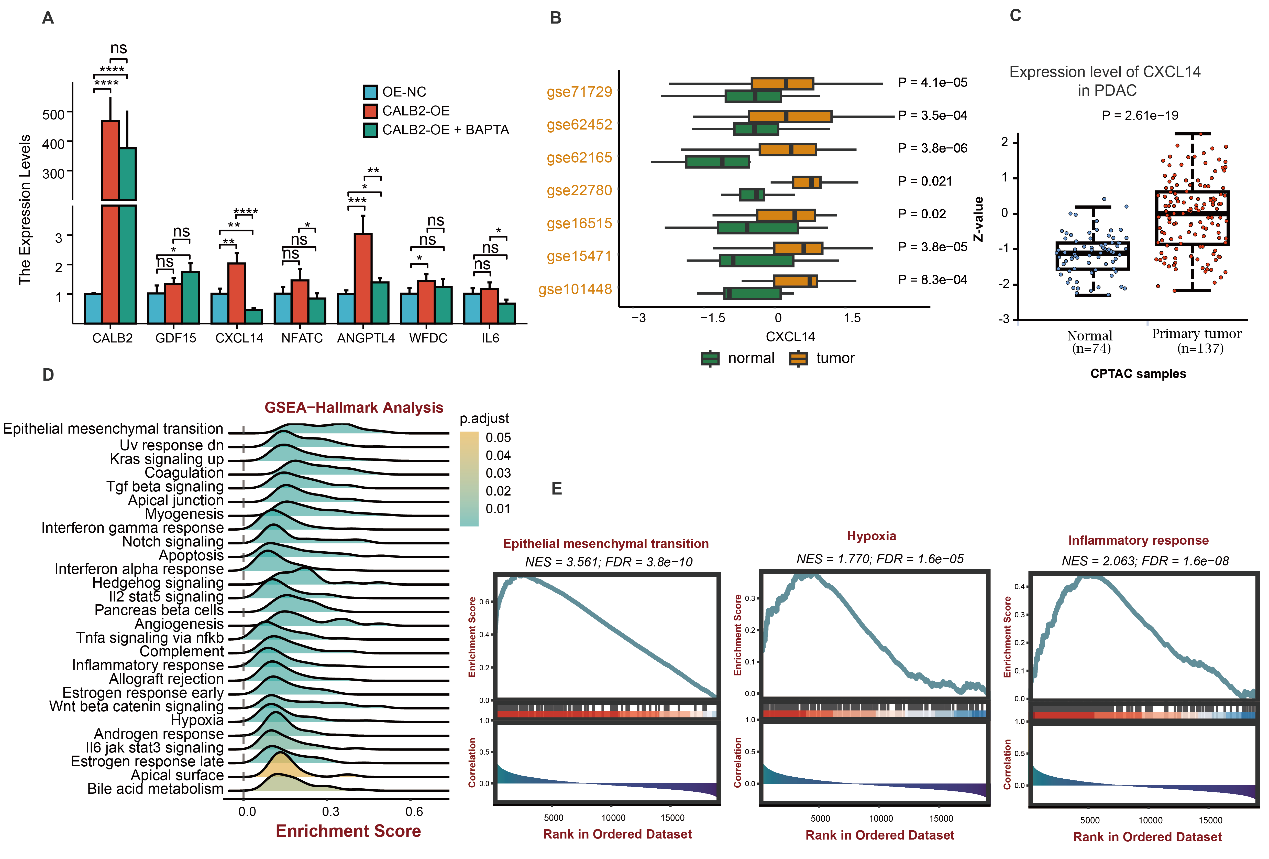
**

**Fig. S7. CXCL14 is notably elevated in PDAC tumor tissues and shares similar enrichment pathways with CALB2. (A)** CALB2-OE BxPC-3 cells were treated with or without 20 μM BAPTA for 48 h, followed by RT-qPCR. **(B)** Comparison of CXCL14 expression in tumor and adjacent normal tissues using multiple PDAC transcriptome data from the GEO. **(C)** Comparison of the protein abundance of CALB2 in PDAC tumor and adjacent normal tissues using the proteomic data from the CPTAC. **(D-E)** Ridge-Plot **(D)** and GSEA-Plot **(E)** for the GSEA-Hallmark enrichment analysis. Error bars, mean ± SD; *p < 0.05, **p < 0.01, ***p < 0.001, ****p < 0.0001; ns, not significant; by one-way ANOVA **(A)** or Student’s t test **(B** and **C)**.
