# Supplementary material for: CALB2 drives pancreatic cancer metastasis through inflammatory reprogramming of the tumor microenvironment
Source: J Exp Clin Cancer Res. 2024 Oct 3;43:277. doi: 10.1186/s13046-024-03201-w (PMC11448066; doi:10.1186/s13046-024-03201-w)
Supplement: Supplementary file 2 — Supplementary Material 2. [file 13046_2024_3201_MOESM2_ESM.docx]

**Supplementary Methods**

**Immunohistochemistry (IHC) staining and sirius red staining**

Thirty-six postoperative PDAC specimens were deparaffinized, rehydrated and boiled for 20 min in a repair box of sodium citrate buffer (pH 6.0) or Tris-EDTA (pH 8.0) antigen retrieval buffer. Endogenous peroxidase activity was blocked by 3% hydrogen for 15 min. After blocked by 5% goat serum for 30 min at room temperature (RT), the tissues were incubated with the following antibody overnight at 4℃: anti-CALB2 (Abcam, #ab92341, 1:200), anti-FAP (Abcam, #ab314456, 1:250), and anti-CK19 (Abcam, #ab52625, 1:100). Then the sections were incubated at RT with HRP-conjugated secondary antibody for 30 min, DAB for 20 s to 1 min, followed by hematoxylin staining. Sirius red staining was conducted using a Picrosirius Red Staining Kit (Polysciences, #24901), according to the manufacturer’s instructions. Staining intensity was assessed by thresholding for positive staining and normalizing to total tissue area, using ImageJ software (NIH).

**RNA extraction and RT-qPCR**

Total RNA was extracted from samples using the RNA-Quick Purification Kit (ES Science, #RN001) and the RNA concentration was measured using the Thermo Scientific™ NanoDrop™ spectrophotometer. The extracted RNA was then reverse transcribed into complementary DNA (cDNA) using the PrimerScript™ RT reagent Kit with gDNA eraser (Takara, #RR047A). Quantitative real‑time PCR (qPCR) was performed using the TB Green^®^ *Premix Ex Taq*™ kit (Takara, #RR420A) on the QuantStudio3 Real-Time PCR System (ThermoFisher Scientific, USA). Glyceraldehyde-3-phosphate dehydrogenase (GAPDH) served as the endogenous control and the relative expression of transcripts was calculated through the 2^−ΔΔCt^ method. All assays were performed according to the manufacturers’ instructions. All used primers are listed in Supplementary Table S3.

**Immunoblotting analysis (IB) and ELISA assay**

Cultured cells were lysed in RIPA buffer (APPLYGEN, #C1053) supplemented with protease inhibitors (AbMole, #M5293) and phosphatase inhibitor (AbMole, #M7528). The total protein concentration was quantified using the Pierce™ BCA Protein Assay Kit (Thermo Fisher Scientific, #UE284362). Total protein lysates were mixed with 5 × loading buffer (GeneStar, #20BB01), separated by SDS-PAGE, and transferred to PVDF membrane. After blocking with TBST containing 3% bovine serum albumin (BSA) for 1 h room temperature, the membrane was incubated with the appropriate primary antibody. Then, the membrane was incubated with horseradish peroxidase (HRP) conjugated goat anti-rabbit or anti-mouse IgG (diluted 1:5000) for 1 h at room temperature. After three extensive washes with TBST for a total of 30 min, the target protein bands were visualized by SuperEnhanced chemiluminescence detection reagents (Applygen, #P0150) in a chemiluminescence gel imager. All the used antibodies and working dilution are detailed in Supplementary Table S5. The concentrations of cytokines in culture media were evaluated using AuthentiKine™ Human IL-6 ELISA Kit (Proteintech, #KE00139), according to the manufacturer’s instructions.

**Co-culture assay of PDAC cells and CAFs**

For the co-culture model of PDAC cells and CAFs, 2×10^5^ CAFs were plated in the upper chamber of transwell apparatus, and 3×10^5^ PDAC cells were cultured in the lower chamber. After incubation for 48-72 h, PDAC cells were collected for RT-qPCR or Western blot. For CAF conditioned medium (CM) preparation, CAFs were cultured to reach 80% confluence and then replaced with serum-free medium for 48 h. The supernatants were collected and centrifuged to remove cell pellets. The CM was used to PDAC cell culture or stored at -80 °C.

For the transwell co-culture assay of migration, PDAC cells (3 × 10^4^/well for PANC-1, 9 × 10^4^/well for BxPC-3/CFPAC-1, and 1.2 × 10^5^/well for AsPC-1) were suspended in serum-free corresponding medium and plated in the upper chambers (Costar, #3422). The lower chamber was seeded with corresponding CAFs (3 × 10^5^/well). After 24 h, the migrated cells on the lower side surface of the filter were fixed by methanol for 20 min and stained with 0.5% crystal violet solution for 20 min. After washing and drying, the number of stained cells of five high-power fields in microscope per chamber filter was calculated. Three independent filters were analyzed per group.

**Cell proliferation and drug resistance assay**

For cell proliferation assay, PDAC cells (2×10^3^/well) were seeded into 96-well plates in triplicate. Subsequently, cell proliferation was evaluated using a Cell Counting Kit-8 (CCK-8) assay (Dojindo, #CK04). The absorbance of each well was measured at 450 nm using a Multiskan SkyHigh Microplate Spectrophotometer (ThermoFisher Scientific, Waltham, MA, USA). For drug resistance assay, PDAC cells (4×10^3^/well) were seeded into 96-well plates in triplicate. A concentration gradient of gemcitabine (Gemzar, Lilly France, dissolved in phosphate-buffered saline [PBS]) was added after cell adhesion. CCK8 was added to cells after 48 h of treatment with gemcitabine. The inhibition rate of different concentration gradient gemcitabine on PDAC cells was calculated according to the following formula: inhibition rate = 1-OD_gem_/OD_control_, where OD_gem_ and OD_control_ represent OD450 value of cells with and without gemcitabine, respectively.

**Mouse experiments**

For the in vivo metastatic assay, 1×10^6^ luciferase-expressing CFPAC-1 or BxPC-3 cells in 50 uL of PBS were injected into the spleen of NSG mice using insulin needles, followed by a 60s pause to avoid reflux and another 2 min press to prevent leakage. In vivo and ex vivo liver bioluminescent signals were measured using an the IVIS Spectrum In Vivo Imaging System. In brief, mice were anesthetized and injected intraperitoneally with 15 mg/mL D-Luciferase potassium salt (10μL/g). Six weeks after injection, the livers were dissected within 7-10 min after substrate injection to evaluate the distant metastasis using ex vivo imaging. The liver metastatic burden was quantified by IVIS Spectrum Living Image software according to the manufacturer’s instructions. Their xenografts were fixed in 4% poly-formaldehyde solution and prepared for H&E examination, or immediately flash-frozen in liquid nitrogen and subsequently used for total RNA extraction.

For the in vivo coinoculation growth assay, 1×10^6^ patient-derived luciferase-expressing PDAC organoids mixed with CAFs at a ratio of 2:1 resuspended in 50 μL FBS-free medium containing 50% Matrigel were injected orthotopically into the pancreas using insulin needles, followed by a 15s pause to avoid reflux and another 2 min press with a cotton swab to prevent leakage. Five weeks after injection, tumor growth and metastasis were evaluated in vivo and ex vivo using the IVIS imaging system. Tumor volumes were calculated using the formula L×W^2^×0.52, where L and W represent length and width, respectively. The primary pancreatic tumor and liver tissues were fixed and excised for H&E examination.

To examine the in vivo metastatic capacity of CALB2-overexpressing PDAC in immunocompetent conditions, stably transfected Calb2-OE or control KPC organoids were generated. These organoids were dissociated to single cells using TrypLE™ Express (Gibco, #12605010), and were then injected into the spleen of syngeneic immunocompetent C57BL/6 mice at an account of 5×10^5^. Tumor metastasis was evaluated in vivo and ex vivo using the IVIS imaging system every 2 weeks. Five weeks after injection, the mice were euthanized, and liver metastases were counted and then excised for H&E examination. Notably, Calb2-OE-LM KPC organoids were established from liver metastases of Calb2-OE KPC organoids as described in Methods.

To evaluate the therapeutic effect of targeting CALB2-CXCL14 axis in immunocompetent conditions, CXCL14 neutralizing antibody is employed 1 week after the intrasplenic injection of Calb2-OE-LM KPC organoids (5×10^5^), when the xenografts was detectable. The mice were randomly divided into four groups and intravenously administered with the IgG control (mouse IgG2A isotype control, R&D Systems, #MAB003) or αCXCL14 (1 mg/kg, R&D Systems, #MAb866), or intraperitoneally injected with PBS control or gemcitabine (25 mg/kg) twice per week for 4 consecutive weeks until the mice were euthanized. Animal survival was plotted as a function of time up to 8 weeks for Kaplan-Meier survival curves.
