# Supplementary material for: CALB2 drives pancreatic cancer metastasis through inflammatory reprogramming of the tumor microenvironment
Source: J Exp Clin Cancer Res. 2024 Oct 3;43:277. doi: 10.1186/s13046-024-03201-w (PMC11448066; doi:10.1186/s13046-024-03201-w)
Supplement: Supplementary file 3 — Supplementary Material 3. [file 13046_2024_3201_MOESM3_ESM.docx]

**Supplementary Table S1-6**

**Supplementary Table S1. shRNA sequences for gene knockdown**

| Gene | Sequences |
| --- | --- |
| Negative Control | 5'-TTCTCCGAACGTGTCACGT-3' |
| shCALB2#1 | 5'-GTCAAAGAGTGACAACTTT-3' |
| shCALB2#2 | 5'-CTCCAGGAATACACCCAAA-3' |
| shCALB2#3 | 5'-TGGGTATATTGAAGGTAAA-3' |
| shCXCL14#1 | 5'-GCAGGGTCTACGAAGAATA-3' |
| shCXCL14#2 | 5'-GCTTCATCAAGTGGTACAA-3' |
| shCXCL14#3 | 5'-AGGAGAAGATGGTTATCAT-3' |
| shSTAT3#1 | 5′-CCCGUCAACAAAUUAAGAATT -3′ |
| shSTAT3#2 | 5′-GCAACAGAUUGCCUGCAUUTT-3′ |
| shSTAT3#3 | 5′-GCGUCCAGUUCACUACUAATT-3′ |

**Supplementary Table S2. sgRNA sequences for CRISPR-Cas9**

| Gene | Sequences |
| --- | --- |
| Negative Control | 5'-CGCTTCCGCGGCCCGTTCAA-3' |
| sgCALB2#1 | 5'-GGGCTCCAGCGCCGAGTTTA-3' |
| sgCALB2#2 | 5'-GCTCGGCCAGGTGCAGGTAA-3' |
| sgCALB2#3 | 5'-GGCGGAAGTACGACACAGAC-3' |

**Supplementary Table S3. Primer sequence for RT-qPCR**

| Gene | Forward primer (5’to 3’) | Reverse primer (5’to 3’) |
| --- | --- | --- |
| GAPDH | GGAGCGAGATCCCTCCAAAAT | GGCTGTTGTCATACTTCTCATGG |
| CALB2 | GCGGAAGTACGACACAGACA | TCTGAGGTCAGCTTCATGCC |
| CXCL14 | GCACTGCGAGGAGAAGATGGTTATC | TTCCAGGCGTTGTACCACTTGATG |
| IL6 | GCCTTCGGTCCAGTTGCCTTC | GTTCTGAAGAGGTGAGTGGCTGTC |
| NFATC2 | GTGCCTGCCATTCCCATCTGC | TCGTAAGAGCCTGACTGACTGGAC |
| ANGPTL4 | CCAAGCCTGCCCGAAGAAAGAG | CGGTTGAAGTCCACTGAGCCATC |
| WFDC2 | TCAGGCACAGGAGCAGAGAAGAC | CATTGGGCAGAGAGCAGAAGGTG |
| GDF15 | TCAGGACGGTGAATGGCTCTCAG | TCGGAATCTGGAGTCTTCGGAGTG |
| ACTA2 | CTTCGTTACTACTGCTGAGCGTGAG | CCCATCAGGCAACTCGTAACTCTTC |
| FAP | GAGTCCAGAATGTTTCGGTCCTGTC | AACAAAGAATCCACCAGCCCATCC |
| CDH1 | GCCATCGCTTACACCATCCTCAG | CTCTCTCGGTCCAGCCCAGTG |
| CDH2 | AGGAGTCAGTGAAGGAGTCAGCAG | TTCTGGCAAGTTGATTGGAGGGATG |
| STAT3 | GGAGGAGTTGCAGCAAAAAG | TGTGTTTGTGCCCAGAATGT |

**Supplementary Table S4. Primers of CALB2 promoter for CUT&RUN-qPCR assay**

| Gene | Forward primer (5’to 3’) | Reverse primer (5’to 3’) |
| --- | --- | --- |
| Promoter1 | GCTCCTGCCAGTTTATCTGC | GTGGTGTAACCTTGGCTGCT |
| Promoter2 | AGAGCCTGCTGGGGTAGTG | ACAGTTGTGCCCAGAGCAG |

**Supplementary Table S5. List of primary antibodies**

| **Antibodies** | **Source** | **Identifier** | **Application** | **Dilution** |
| --- | --- | --- | --- | --- |
| Rabbit polyclonal anti-CALB2 (clone EP1798) | Abcam | Cat# ab92341; RRID:AB_2049245 | WB; IHC; IF | 1:1000; 1:200; 1:100 |
| Rabbit polyclonal anti-FAP (clone RM1080) | Abcam | Cat# ab314456; RRID: AB_3097779 | WB; IHC; IF | 1:1000; 1:250; 1:100 |
| Rabbit monoclonal anti-Cytokeratin 19 (clone EP1580Y) | Abcam | Cat# ab52625; RRID: AB_2281020 | IHC; IF | 1:100; 1:100 |
| Rabbit monoclonal anti-PD-L1 (clone E1L3N) | Cell Signaling Technology | Cat# 13684; RRID: AB_2687655 | IHC | 1:200 |
| Rabbit monoclonal anti- Vinculin | Proteintech | Cat# 66305-1-Ig; RRID: AB_2810300 | WB | 1:5000 |
| Rabbit polyclonal anti- NF-κB p65 | Proteintech | Cat# 10745-1-AP; RRID: AB_2178878 | WB | 1:1000 |
| Rabbit polyclonal anti-Phospho-NF-κB p65 (Ser468) | Proteintech | Cat# 82335-1-RR; RRID: AB_3083091 | WB | 1:2000 |
| Rabbit polyclonal anti- smooth muscle actin | Proteintech | Cat# 14395-1-AP; RRID: AB_2223009 | WB | 1:1000 |
| Rabbit polyclonal anti- IL-6 | Proteintech | Cat# 21865-1-AP; RRID: AB_11142677 | WB | 1:1000 |
| Rabbit polyclonal anti- Beta Tubulin | Proteintech | Cat# 10068-1-AP; RRID: AB_2303998 | WB | 1:1000 |
| Mouse monoclonal anti-  Stat3 (124H6) | Cell Signaling Technology | Cat# 9139; RRID: AB_331757 | WB; CUT&RUN | 1:1000; 1:100 |
| Rabbit monoclonal anti-  Phospho-Stat3 (Tyr705) (D3A7) | Cell Signaling Technology | Cat# 9145, RRID: AB_2491009 | WB | 1:2000 |
| Rabbit monoclonal anti-  Tri-Methyl-Histone H3 (Lys4) (C42D8) | Cell Signaling Technology | Cat# 9751, RRID: AB_2616028 | WB; CUT&RUN | 1:1000; 1:50 |
| Rabbit polyclonal anti- NFATC2 | Proteintech | Cat# 22023-1-AP, RRID: AB_2878973 | WB | 1:2000 |
| Rabbit polyclonal anti-CXCL14 | Affinity Biosciences | Cat# DF12377, RRID: AB_2845182 | WB; IHC | 1:1000; 1:100 |
| Rabbit polyclonal anti- E-cadherin | Proteintech | Cat# 20874-1-AP, RRID: AB_10697811 | WB | 1:20000 |
| Rabbit polyclonal anti- N-cadherin | Proteintech | Cat# 22018-1-AP, RRID: AB_2813891 | WB | 1:2000 |
| Rabbit polyclonal anti- ANGPTL4 | Proteintech | Cat# 18374-1-AP, RRID: AB_2878539 | WB | 1:1000 |

**Supplementary Table S6. Predicted binding sites in the CALB2 promoter region**

| **Matrix ID** | **Name** | **Score** | **Relative score** | **Start** | **End** | **Strand** | **Predicted sequence** |
| --- | --- | --- | --- | --- | --- | --- | --- |
| [MA0144.2](https://jaspar.genereg.net/matrix/MA0144.2) | MA0144.2.STAT3 | 6.237392 | 0.873118 | 820 | 830 | + | CCTCTTGGAAG |
| [MA0144.2](https://jaspar.genereg.net/matrix/MA0144.2) | MA0144.2.STAT3 | 6.020298 | 0.870488 | 191 | 201 | - | ATGCTTGTAAT |
| [MA0144.2](https://jaspar.genereg.net/matrix/MA0144.2) | MA0144.2.STAT3 | 5.640163 | 0.865883 | 1029 | 1039 | + | CTGCTGGGGAG |
| [MA0144.2](https://jaspar.genereg.net/matrix/MA0144.2) | MA0144.2.STAT3 | 5.282355 | 0.861548 | 342 | 352 | - | ATGCCTGTAAT |
| [MA0144.2](https://jaspar.genereg.net/matrix/MA0144.2) | MA0144.2.STAT3 | 4.751634 | 0.855119 | 404 | 414 | - | CTTCTGCAAAA |
| [MA0144.2](https://jaspar.genereg.net/matrix/MA0144.2) | MA0144.2.STAT3 | 4.496147 | 0.852024 | 618 | 628 | + | GTTCCTGCAAG |
| [MA0144.2](https://jaspar.genereg.net/matrix/MA0144.2) | MA0144.2.STAT3 | 4.422844 | 0.851136 | 468 | 478 | + | ATTCTGGGATT |
| [MA0144.2](https://jaspar.genereg.net/matrix/MA0144.2) | MA0144.2.STAT3 | 3.026844 | 0.834224 | 1713 | 1723 | + | CTGCCAGCAAT |
| [MA0144.2](https://jaspar.genereg.net/matrix/MA0144.2) | MA0144.2.STAT3 | 2.852054 | 0.832106 | 247 | 257 | - | CTACTAAAAAA |
| [MA0144.2](https://jaspar.genereg.net/matrix/MA0144.2) | MA0144.2.STAT3 | 2.389389 | 0.826501 | 334 | 344 | + | GTGCTAGGATT |
